# Mitochondrial succinate dehydrogenase function is essential for sperm motility and male fertility

**DOI:** 10.1016/j.isci.2022.105573

**Published:** 2022-11-14

**Authors:** Rachel M. Woodhouse, Natalya Frolows, Guoqiang Wang, Azelle Hawdon, Edmund Heng Kin Wong, Linda C. Dansereau, Yingying Su, Liam D. Adair, Elizabeth J. New, Ashleigh M. Philp, Wei Kang Tan, Andrew Philp, Alyson Ashe

**Affiliations:** 1 The University of Sydney, School of Life and Environmental Sciences, Sydney, NSW 2006, Australia; 2 Division of Genome Science and Cancer, The John Curtin School of Medical Research, The Australian National University, Canberra, ACT 2601, Australia; 3 CSIRO Health and Biosecurity, Sydney, NSW 2113, Australia; 4 Department of Molecular Biology and Biochemistry, Nelson Biological Laboratories, Rutgers, The State University of New Jersey, Piscataway, NJ 08854, USA; 5 Australian Regenerative Medicine Institute, Monash University, Clayton, VIC 3800, Australia; 6 Garvan Institute of Medical Research, Sydney, NSW 2010, Australia; 7 St Vincent’s Clinical School, UNSW Medicine, University of NSW, Sydney, NSW 2010, Australia; 8 Sydney Microscopy and Microanalysis, The University of Sydney, Sydney, NSW 2006, Australia; 9 The University of Sydney, School of Chemistry, Sydney, NSW 2006, Australia; 10 Australian Research Council Centre of Excellence for Innovations in Peptide and Protein Science, The University of Sydney, Sydney, NSW 2006, Australia; 11 Centre for Healthy Ageing, Centenary Institute, Missenden Road, Sydney, NSW 2050, Australia; 12 Charles Perkins Centre, Faculty of Medicine and Health, University of Sydney, NSW 2006, Australia

**Keywords:** Biological sciences, biochemistry, molecular biology, cell biology

## Abstract

Mitochondrial health is crucial to sperm quality and male fertility, but the precise role of mitochondria in sperm function remains unclear. SDHA is a component of the succinate dehydrogenase (SDH) complex and plays a critical role in mitochondria. In humans, SDH activity is positively correlated with sperm quality, and mutations in SDHA are associated with Leigh Syndrome. Here we report that the *C. elegans* SDHA orthologue SDHA-2 is essential for male fertility: *sdha-2* mutants produce dramatically fewer offspring due to defective sperm activation and motility, have hyperfused sperm mitochondria, and disrupted redox balance. Similar sperm motility defects in *sdha-1* and *icl-1* mutant animals suggest an imbalance in metabolites may underlie the fertility defect. Our results demonstrate a role for SDHA-2 in sperm motility and male reproductive health and establish an animal model of SDH deficiency-associated infertility.

## Highlights

• Loss of SDHA-2 causes male infertility
• Sperm in *sdha-2* mutants fail to activate and are largely immotile
• Sperm defects correlate with mitochondrial abnormalities and disrupted redox balance
• Occasional revertants have normal fertility, suggesting infertility can be rescued

## Graphical abstract

**Figure:**
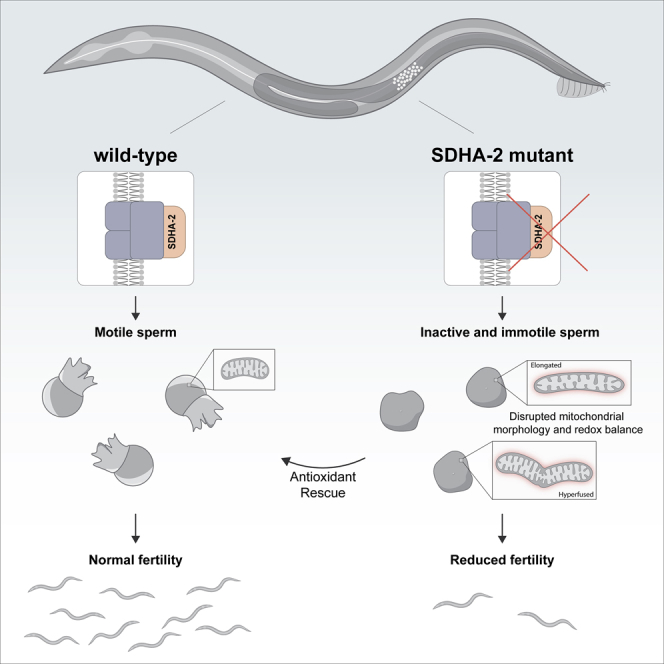

## Introduction

Approximately 1 in 20 men currently experience reduced fertility which causes personal, societal, and economic burdens.^1^^,^^2^^,^^3^ Semen quality is essential to fertility and can be measured using metrics such as sperm count, morphology, DNA quality, and motility. Sperm from infertile males have decreased mitochondrial volume and disordered mitochondria, and research has implicated mitochondrial function in sperm quality, particularly in sperm motility.^4^

A major function of mitochondria is the generation of ATP via oxidative phosphorylation. In this process, electrons are derived from the oxidation of NADH or succinate and transported through the electron transport chain (ETC), a series of four enzymatic complexes termed Complex I-IV. Various aspects of oxidative phosphorylation have been linked to sperm function in mammals,^5^^,^^6^ and exposure to ETC inhibitors results in decreased sperm motility.^7^^,^^8^ Oxygen is consumed in the final step of the ETC, and the mitochondrial oxygen consumption rate is positively correlated with sperm motility.^9^^,^^10^ There is also a positive association between mitochondrial membrane potential and sperm function.^4^

Besides ATP production, mitochondria have a range of other functions including the generation of metabolites in the tricarboxylic acid (TCA) cycle. These metabolites have diverse downstream applications, including in signaling, epigenetic regulation, and as intermediates in the synthesis of pyrimidine, purine, amino acids, and fatty acids.^11^ The link between TCA cycle activity and metabolite homeostasis in sperm function is unclear. Therefore, while the evidence clearly supports an association between mitochondrial function and sperm health, the precise role of mitochondria in sperm function and motility is still unclear.

Succinate dehydrogenase (SDH) or ETC Complex II is an enzyme complex that exists at the nexus of the ETC and TCA cycles, essential to the function of both: it converts succinate to fumarate in the TCA cycle, and this reaction contributes electrons to the ETC. SDHA is the catalytic subunit of the SDH complex. In the most severe cases, SDHA deficiency in humans causes Leigh syndrome, a devastating disorder that arises from defects in oxidative phosphorylation or related processes which contribute to mitochondrial energy production.^12^ Representing more mild cases, a variant in SDHA has been associated with male infertility in humans,^13^ and sperm from infertile males display decreased and dispersed SDH localization.^14^

Deletion of mitochondrial factors is almost always homozygous lethal, so in this work, we have taken advantage of a gene duplication in *Caenorhabditis. elegans,* which have two orthologues of SDHA; SDHA-1 and SDHA-2. *sdha-1* homozygous mutants are non-viable, but *sdha-2* homozygous mutants are viable. Furthermore, SDHA-2 is highly homologous to human SDHA, with the two proteins sharing 71% amino acid sequence identity. As eukaryotes, *C. elegans’* ETC and TCA cycle proteins are highly homologous to their mammalian counterparts and their sperm contain many mitochondria.^15^ Thus, *C. elegans* SDHA-2 provides an ideal opportunity to study the role of both the ETC and TCA cycle in sperm function.

*C. elegans* populations primarily comprise self-fertilizing hermaphrodites which produce both oocytes and self-sperm but also include male sperm-only animals. Spermiogenesis is the process by which spermatids mature into functional spermatozoa capable of motility. Mammalian sperm assemble a flagellum which allows them to “swim.” Like mammalian sperm, *C. elegans* sperm mature from immotile spermatids into motile spermatozoa in a process called sperm activation.^16^ Rather than the flagellum of mammalian sperm, mature *C. elegans* sperm have a pseudopod and move by “crawling.”^17^^,^^18^ Motility is driven by the polymerization of major sperm protein in a system somewhat analogous to the actin-driven motility of most other eukaryotic ameboid cells.^19^

In this study, we demonstrate that SDHA-2 is critical for male fertility. We show that *sdha-2* mutants have defective male sperm and hermaphrodite self-sperm which fail to activate into motile spermatozoa. We implicate metabolite homeostasis and oxidative stress in *sdha-2* mutant sperm dysfunction, expanding our understanding of the role of the ETC and TCA cycle in sperm health. We also describe rare incidences of reversion to normal fertility in *sdha-2* mutants and identify stress-responsive pathways intimately connected to SDHA-2 function which could mediate this rescue. These findings suggest the potential for novel avenues of treatment for SDH deficiency-related pathologies.

## Results

### *sdha-2* mutants have a fertility defect

**Figure 1:**
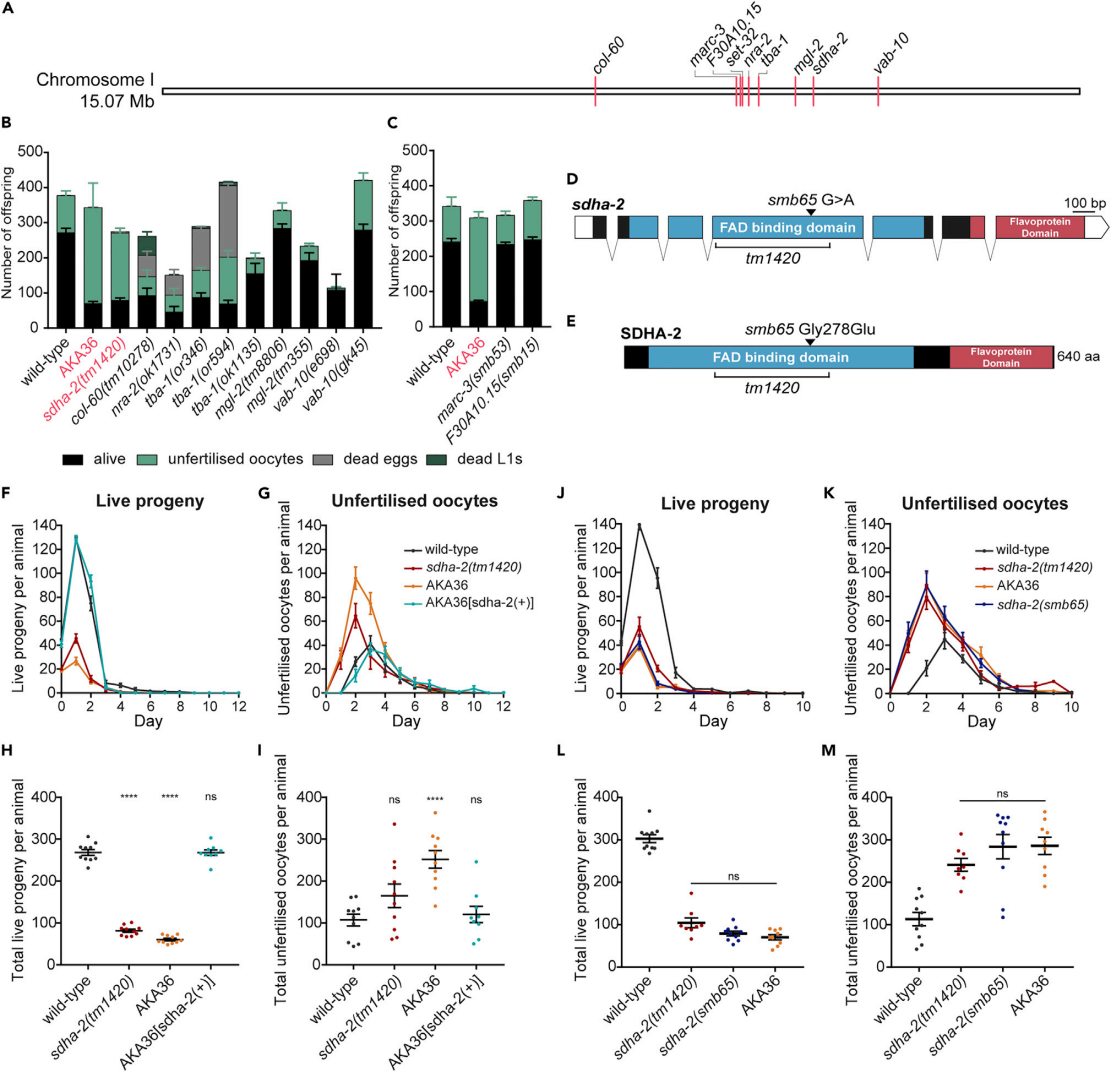
*sdha-2* mutant animals display a brood size defect (A) Whole-genome sequencing of AKA36 revealed 9 non-synonymous mutations on Chromosome I, in addition to the previously characterized *set-32(ok1457)* deletion. (B and C) Brood size screen of the candidate genes identified by AKA36 whole genome sequencing. (B) Publicly available strains. (C) Strains created by CRISPR-Cas9, recreating the mutations from AKA36 in the wild-type background. Data are mean ± SEM; n = 5-10. (D) Schematic representing the *sdha-2* transcript. Exons are represented by black boxes, introns by connecting lines, and untranslated regions by white boxes. bp denotes length in base pairs. (E) Predicted SDHA-2 protein. aa denotes length in amino acids. (D and E) The sequence encoding the FAD binding domain is indicated in blue and the succinate dehydrogenase/fumarate reductase flavoprotein C-terminal domain (flavoprotein domain) is in maroon. AKA36 carries a Gly > Glu substitution in SDHA-2 at position 278 (arrow). *sdha-2(tm1420)* has a 173 aa in-frame deletion (bracket). (F–I) Brood size assay characterizing *sdha-2* mutant infertility. (F and G) The average number of (F) live progeny and (G) unfertilized oocytes produced per animal per day. (H and I) The total number of (H) live progeny and (I) unfertilized oocytes per animal. AKA36[*sdha-2(+)*] was generated by repairing the *sdha-2* SNV in AKA36 back to the wild-type sequence by CRISPR-Cas9. Data are mean ± SEM; n = 9-10. Comparisons are between the indicated strain and wild-type and were performed using one-way ANOVA with Dunnett’s post hoc test, ∗∗∗∗p ≤ 0.0001. (J–M) Brood size assay comparing different *sdha-2* mutations. (J and K) The average number of (J) live progeny and (K) unfertilized oocytes produced per animal per day. (L and M) The total number of (L) live progeny and (M) unfertilized oocytes per animal. Data are mean ± SEM; n = 9-10. Comparisons were performed using one-way ANOVA with Tukey’s post hoc test.

We previously reported that *C. elegans* strain AKA36 has a male fertility defect.^20^ AKA36 harbors the 514 bp deletion *set-32(ok1457)* on chromosome I, and was outcrossed six times prior to our previous study. To further investigate the defect, we performed whole genome sequencing on AKA36 and wild-type worms. We identified nine non-synonymous homozygous mutations on chromosome I present in AKA36, in addition to the previously characterized *set-32(ok1457)* deletion (Figure 1A). Eight of the nine were single nucleotide variations (SNV), and one was a five-base pair deletion (Table S1). One of the nine mutations was an additional mutation in *set-32*, while the remaining eight were in other genes. Mutant strains were publicly available for six of the eight genes. For the remaining two genes, we used CRISPR/Cas9 to recreate the AKA36 mutation identified by our whole genome sequencing in wild-type worms.

We performed brood size assays on self-fertilized hermaphrodite animals to test whether any of these other genes were responsible for the fertility defect. Mutations in seven of the eight genes produced different proportions of live progeny and unfertilized oocytes to AKA36 (Figures 1B and 1C). However, *sdha-2(tm1420)* animals displayed a strikingly similar brood size defect to AKA36 animals (Figure 1B), producing significantly less live progeny than the wild type, and a similar but not significant trend of increased unfertilized oocytes (Figures 1F–1I). AKA36 and *sdha-2* mutants also displayed striking similarities in the temporal distribution of live progeny and unfertilized oocyte production (Figures 1F and 1G). Both strains exhibited depressed live progeny production in the first two days of adulthood compared with wild-type, and early-onset unfertilized oocyte production from day 1, peaking at day 2.

*sdha-2(tm1420)* has a 173 aa in-frame deletion in the SDHA-2 FAD binding domain (position 135-307, with an Ala > Ser substitution at the deletion site) (Figures 1D and 1E). The SNV in *sdha-2* in strain AKA36 (that we named *sdha-2(smb65))* is a missense mutation: a G to A mutation in exon 4 results in a Gly > Glu substitution at position 278 in the FAD binding domain (Figures 1D and 1E). To confirm that the *sdha-2(smb65)* SNV is responsible for the fertility defect, we used CRISPR-Cas9 to repair the SNV in AKA36 back to the wild-type sequence (referred to hereafter as AKA36[*sdha-2(+)*]). The repair completely rescued both live progeny and unfertilized oocyte numbers to wild-type levels (Figures 1F–1I). We also recreated *sdha-2(smb65)* in a wild-type background. This strain displayed the same fertility defect as AKA36 and *sdha-2(tm1420)* (Figures 1J–1M). Therefore, we concluded that the *sdha-2* SNV in strain AKA36 is responsible for the observed brood size defect.

**Figure 2:**
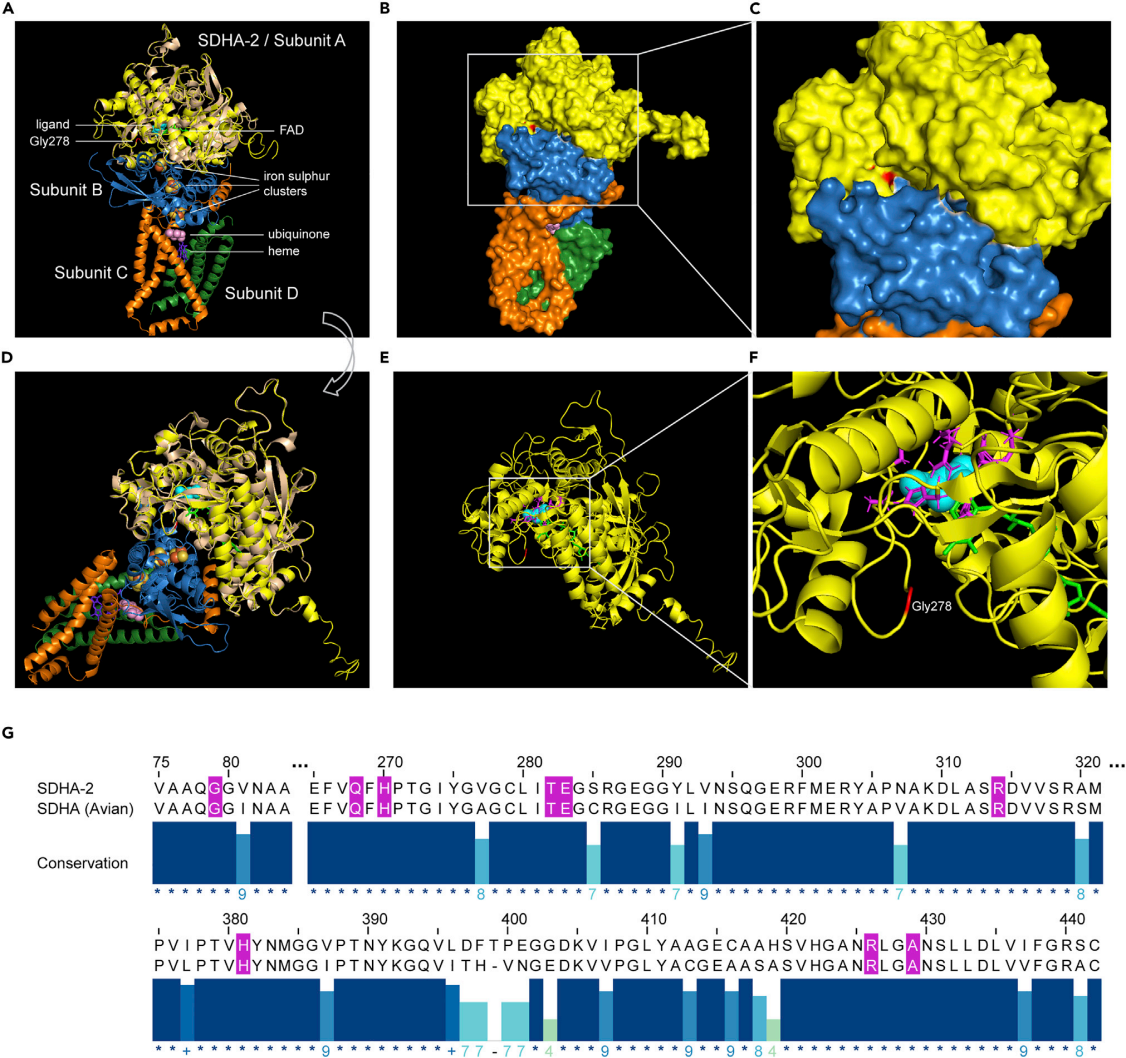
SDHA-2 protein model (A–F) I-TASSER-generated model of SDHA-2. (A–C) show the complex from a perspective highlighting the 4 subunits; (D–F) show the protein/complex from a different perspective highlighting the ligand binding site. The SDHA-2 model is colored yellow and SDHA-2 Gly278 is colored red. (A and D) Two perspectives of the SDHA-2 model aligned with PDB structure PDB: 1YQ3, Avian respiratory complex II with oxaloacetate and ubiquinone (subunits A-D) (Ref.^22^) 1YQ3 Subunit A is beige, B is blue, C is orange, and D is green. Subunit A binds a malate-like ligand (cyan balls) and FAD cofactor (green sticks). Subunit B is an iron-sulphur protein binding three different iron-sulphur clusters (yellow and orange balls). Subunits C and D bind a heme molecule (purple sticks). Ubiquinone is represented by pink balls. (B and C) Surface model of SDHA-2 and 1YQ3. SDHA-2 Gly278 (red) is near the predicted interface with subunit B (blue). (E and F) The SDHA-2 model alone. Side chains of the residues comprising the predicted malate-like ligand binding site (from homology with Avian respiratory complex II subunit A (Ref.^22^) are colored magenta. (G) Amino acid sequence alignment of regions of SDHA-2 to Avian SDHA using Clustal Omega. Numbering refers to SDHA-2 residues. Residues comprising the predicted malate-like ligand binding site are colored magenta (Ref.^22^)

Both the *smb65* SNV and *tm1420* large deletion result in dramatic fertility defects (Figures 1J–1M). To investigate why a Gly278Glu single amino acid substitution would have such a deleterious effect we modeled *C. elegans* SDHA-2 using the 3D protein structure prediction tool I-TASSER^21^ (Figures 2A–2G). Gly278 is located in a core region of the protein and could disrupt the predicted interface with complex II subunit B (Figures 2B and 2C) and/or the predicted malate-like intermediate ligand binding site and FAD cofactor binding site (Figures 2E–2G).

### *sdha-2* mutants have defective hermaphrodite self-sperm and male sperm

**Figure 3:**
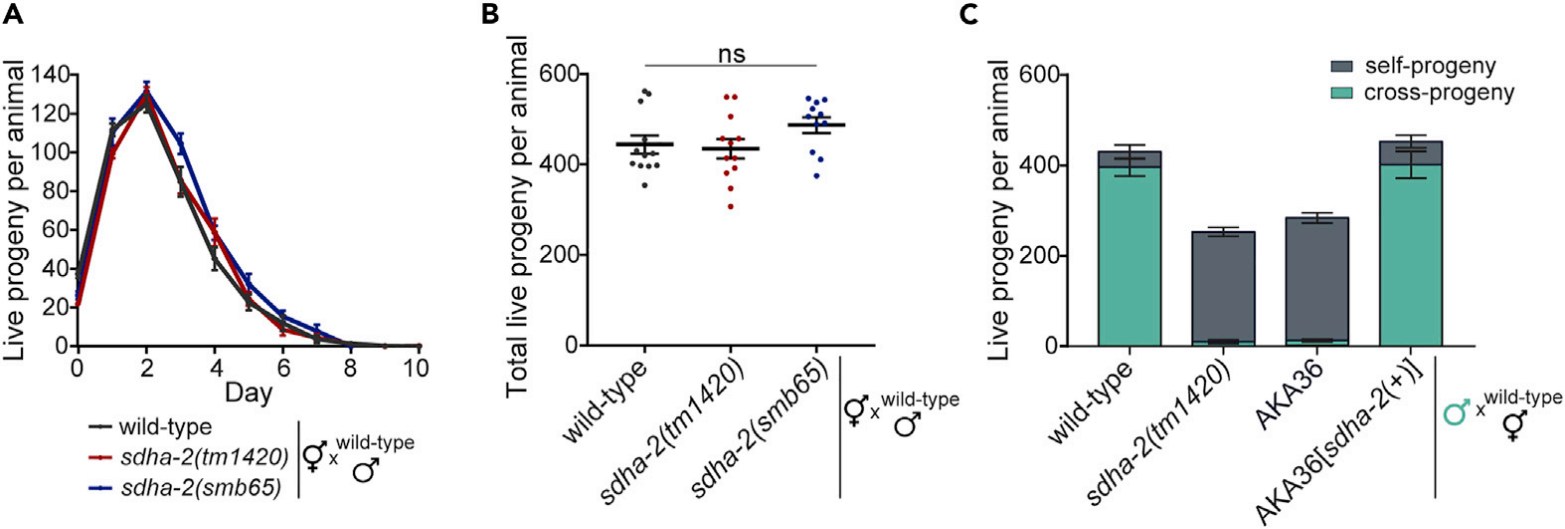
*sdha-2* mutants have defective hermaphrodite self-sperm and male sperm (A and B) Brood size assay of wild-type, *sdha-2(tm1420),* or *sdha-2(smb65)* hermaphrodites mated to wild-type males. (A) The average number of live progeny produced per mated animal per day. (B) Total number of live progeny per mated animal. Data are mean ± SEM; n = 11-12. Comparisons were performed using one-way ANOVA with Tukey’s post hoc test. (C) Brood size assay of wild-type hermaphrodites mated to wild-type, *sdha-2(tm1420)*, AKA36, or AKA36[*sdha-2(+)*] (AKA36 with repaired *sdha-2*) males. Males were marked with an integrated *glr-1::gfp* neuronal reporter transgene to distinguish cross-progeny (GFP-positive) from self-progeny (GFP-negative). Data are mean ± SEM; n = 7-12.

The presence of large numbers of unfertilized oocytes from hermaphrodite mutant strains suggests a sperm defect. We previously showed that AKA36 mutants have defective hermaphrodite self-sperm and male sperm.^20^ We sought to recapitulate these findings using the *sdha-2(tm1420)* and *sdha-2(smb65)* alleles. We mated wild-type males with *sdha-2(tm1420)*, *sdha-2(smb65)* or wild-type hermaphrodites. We observed no difference in the resulting brood size compared with wild-type mated brood size (Figures 3A and 3B). Thus, the provision of wild-type male sperm rescues *sdha-2* hermaphrodite infertility, suggesting that oogenesis is unaffected by loss of SDHA and that defective hermaphrodite self-sperm are the cause of the fertility defect.

To investigate male sperm, we mated wild-type males, *sdha-2(tm1420)* males, AKA36 males, and AKA36[*sdha-2(+)*] (AKA36 with repaired *sdha-*2) males each marked with a GFP transgene to wild-type hermaphrodites for 24 h and counted cross-progeny (GFP-positive) and self-progeny (GFP-negative). Wild-type mated controls produced predominantly cross-progeny (Figure 3C) as expected^18^ (mean 396 cross-progeny, 34 self-progeny). In contrast, broods resulting from wild-type hermaphrodites mated to AKA36 or *sdha-2(tm1420)* males comprised primarily self-progeny with very few cross-progeny (mean 11 cross-progeny, 271 self-progeny; 10 cross-progeny, 243 self-progeny respectively), indicating a *sdha-2* mutant male sperm defect. The few cross-progeny produced were laid in parallel with self-progeny during the first few days of adulthood (data not shown), suggesting that mutant male sperm were outcompeted by wild-type hermaphrodite self-sperm. AKA36[*sdha-2(+)*] males mated to wild-type hermaphrodites produced predominantly cross-progeny, mirroring the wild-type male phenotype (Figure 3D). Together, these data demonstrate that loss of functional SDHA-2 results in defective hermaphrodite and male sperm.

### Loss of *sdha-2* causes sperm activation and motility defects

**Figure 4:**
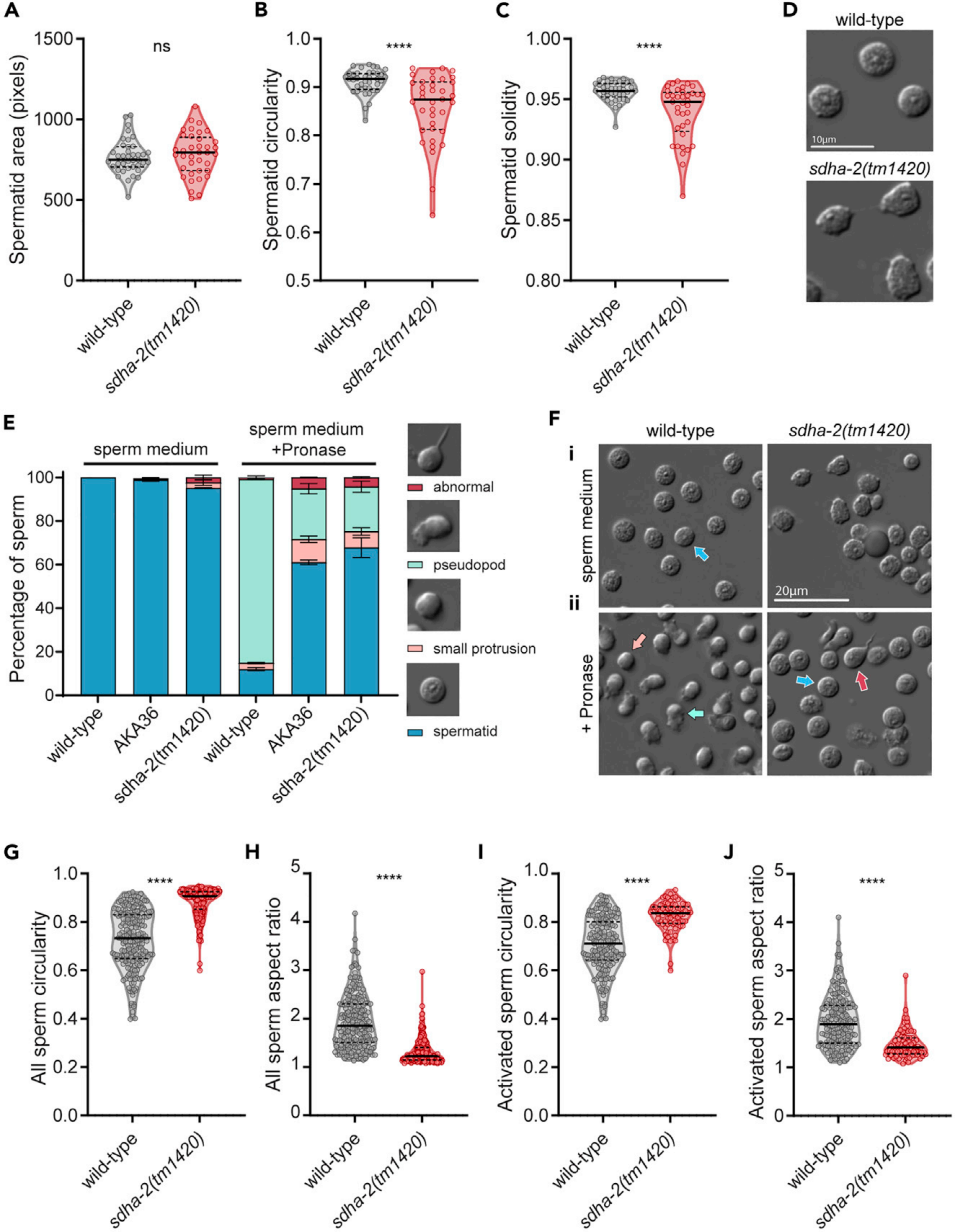
*sdha-2* mutant sperm are defective due to failure to activate into motile spermatozoa (A–D) Virgin adult males were dissected in sperm medium and imaged, and spermatid morphology metrics were quantified using ImageJ: (A) area, (B) circularity (where 1 is a perfect circle), and (C) solidity (where 1 is a completely convex shape). n = 37-39 spermatids from 3 males. Comparisons were performed using an unpaired t test, ∗∗∗∗p ≤ 0.0001. (D) Representative spermatids of the indicated genotype. Scale bar = 10 μm. (E) *In vitro* sperm activation assay. Virgin adult males were dissected in sperm medium with or without Pronase, as indicated. Data are mean ± SEM, n = 2 independent replicates, each comprising >180 sperm from ≥5 males. (F) Representative images of sperm in (i) sperm medium or (ii) sperm medium with Pronase. Arrows mark representative spermatids (blue), spermatids with small protrusion (pink), spermatids with pseudopod (green), and abnormal spermatids (spiked, swollen, or vacuolated) (red). Scale bar = 20 μm. (G–J) Morphology metrics of Pronase-exposed sperm were quantified using ImageJ, including (G and I) circularity and (H and J) aspect ratio (major axis/minor axis). (G and H) All Pronase-exposed sperm, n = 210-211 from ≥5 males. (I and J) Activated spermatozoa in the Pronase-exposed condition, n = 185 for wild-type, 76 for *sdha-2(tm1420)*. Violin plots display the median (solid line) and quartiles (dashed lines). Comparisons were performed using an unpaired t test, ∗∗∗∗p ≤ 0.0001.

The defect in both hermaphrodite self-sperm and male sperm suggested an intrinsic sperm defect. We previously showed that the number of sperm per spermatheca 12 h after L4 in AKA36 and wild-type hermaphrodites is comparable,^20^ indicating that the fertility defects were not due to decreased sperm quantity. To investigate sperm further, we examined isolated spermatids from dissected males. We observed no significant difference in size between wild-type and *sdha-2(tm1420)* spermatids, but the latter had misshapen morphology, characterized by significantly reduced circularity and solidity compared with wild-type (Figures 4A–4D).

Spermiogenesis is the process by which spermatids mature into functional spermatozoa capable of motility, termed sperm activation in *C. elegans*. In order to quantify spermatid activation we supplemented isolated spermatids with Pronase, a broad-acting mix of proteases that promotes *in vitro* sperm activation.^23^ Activated *C. elegans* spermatozoa are identified by the presence of a pseudopod, required for motility (analogous to mammalian flagellum). 85% of wild-type spermatids activated into spermatozoa, as expected (Figures 4E and 4F) while only 23% of *sdha-2(tm1420)* spermatids activated. 63% remained as spermatids, and the remainder grew a small protrusion (9%) or appeared abnormal—vacuolated, swollen, or with an intermediate spiked pseudopod (5%) (Figures 4E and 4F). Similarly, 21% of AKA36 spermatids activated, 62% remained as spermatids, 12% grew a small protrusion and 5% appeared abnormal. Similar activation ratios were observed when spermatids were treated with monensin (Figure S1), a Na^+^ ionophore that also promotes *in vitro* sperm activation.^16^ This suggests that *sdha-2* mutant infertility is due to sperm activation failure.

Pronase-exposed *sdha-2(tm1420)* sperm were significantly more circular than wild-type sperm and had a significantly smaller aspect ratio (Figures 4G and 4H) consistent with a failure to activate and grow a pseudopod. We repeated these analyses on the activated subset of sperm only and observed the same trends (Figures 4I and 4J). This indicates that the small proportion of *sdha-2(tm1420)* sperm that do successfully activate still have reduced pseudopod length and/or extension.

Together, these results demonstrate that most *sdha-2* mutant sperm fail to activate and remain as immotile spermatids, and the small proportion that successfully activate have shortened pseudopods which may result in impaired motility compared with mature wild-type sperm.

**Figure 5:**
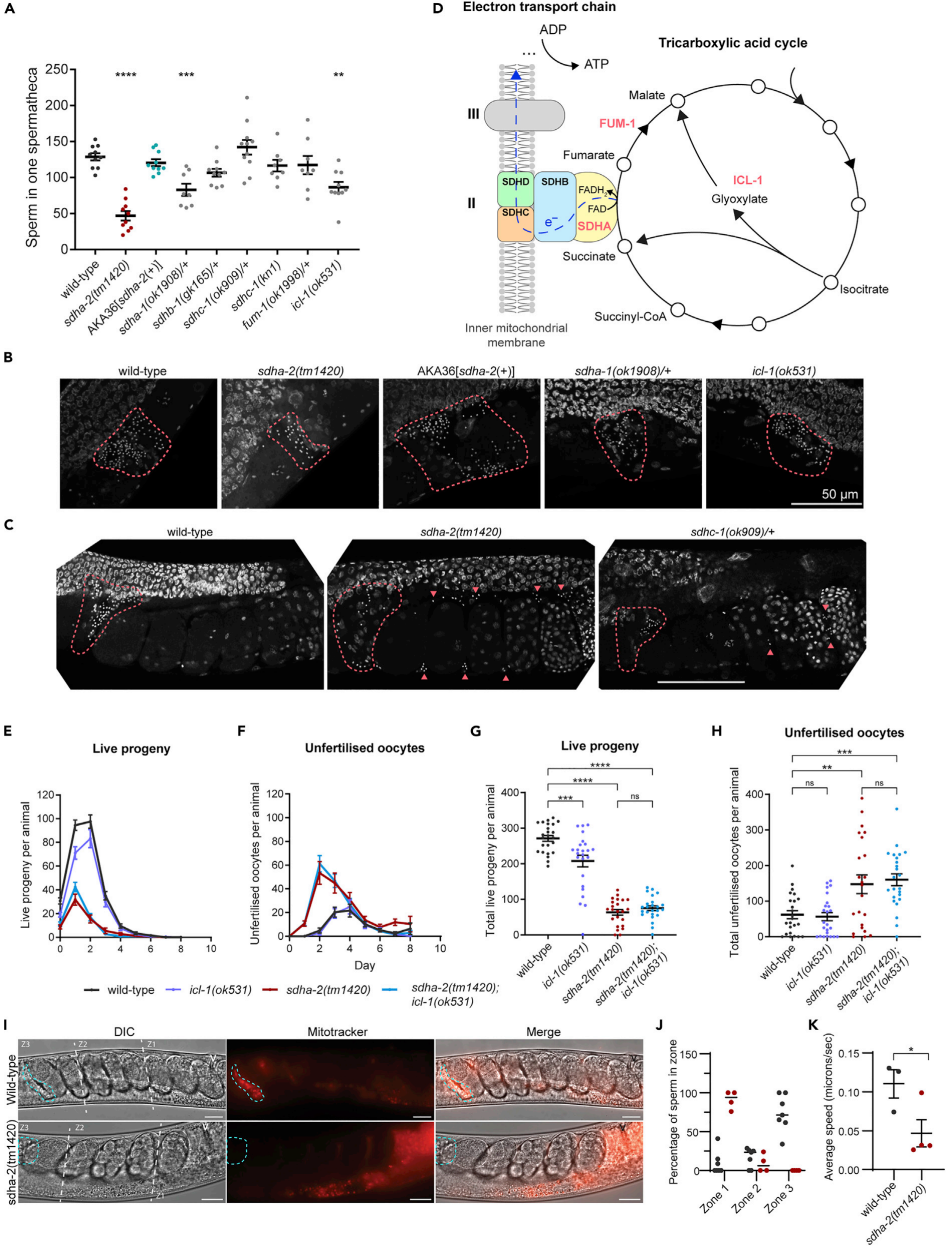
*sdha-1, sdha-2* and *icl-1* mutants have fewer sperm in hermaphrodite spermatheca (A) Number of sperm in one hermaphrodite spermatheca at day 1 of adulthood. Genotype/+ indicates balanced strains, where sperm were counted in a heterozygote hermaphrodite. Data are mean ± SEM; n = 8-11. Comparisons are between the indicated strain and wild-type and were performed using one-way ANOVA with Dunnett’s post hoc test ∗∗p ≤ 0.01, ∗∗∗p ≤ 0.001, ∗∗∗∗p ≤ 0.0001. (B) Representative maximum intensity *Z-*projections of a spermatheca in DAPI-stained day 1 adult hermaphrodites. (C) Representative images of DAPI-stained day 1 adult hermaphrodites. Sperm can be detected throughout the uterus of *sdha-2(tm1420)* and *sdhc-1(ok909)* animals, indicated by arrowheads. (B and C) The spermatheca is outlined in pink. Scale bar = 50 μm. (D) SDH is comprised of four subunits: SDHA, SDHB, SDHC, and SDHD. In *C. elegans,* these are encoded by *sdha-1* and *-2*, *sdhb-1, sdhc-1,* and *sdhd-1* respectively. FUM-1 is the *C. elegans* fumarase, and ICL-1 is the *C. elegans* isocitrate lysase/malate synthase. (E–H) Brood size assay to assess *icl-1* fertility. (E and F) The average number of (E) live progeny and (F) unfertilized oocytes produced per animal per day. (G and H) The total number of (G) live progeny and (H) unfertilized oocytes per animal. All animals were derived from a single cross between *sdha-2(tm1420)* and *icl-1(ok531)* to ensure a consistent genetic background between strains. Three lines were selected from the single cross for each genotype, used in three independent brood size assays (n = 8 worms per assay). Data are mean ± SEM; n = 24. Comparisons were performed using one-way ANOVA with Tukey’s post hoc test ∗∗p ≤ 0.01, ∗∗∗p ≤ 0.001, ∗∗∗∗p ≤ 0.0001. (I) Representative images of MitoTracker Red sperm in the uterus of wild-type hermaphrodites. The spermatheca is outlined in blue and V = vulva. Scale bar = 20 μm. (J) The percentage of sperm in the three zones of the hermaphrodite uterus 1 h after mating (zone 1 = vulval region, zone 2 = central zone, zone 3 = spermathecal region). n = 11 per strain. (K) All visible sperm in zone 2 were tracked and averaged to give the average sperm velocity per gonad arm 30 min-1 hr post-mating. n = 3 (wild-type) and n = 4 (*sdha-2(tm1420)*) gonad arms.

We then visualized sperm in day one adult hermaphrodites by DAPI staining. *sdha-2(tm1420)* animals had significantly fewer sperm per spermatheca than wild-type (Figures 5A and 5B). Sperm were detected throughout the uterus of *sdha-2(tm1420)* mutants, while in wild type all sperm resided in the spermatheca (Figure 5C). In normal hermaphrodite fertilization, oocytes pass through the spermatheca and push sperm into the uterus.^18^ Sperm rapidly migrate back to the spermatheca via the pseudopod which grants motility.^18^ The presence of sperm in the uterus suggests that many *sdha-2(tm1420)* hermaphrodite self-sperm have defective motility.

To quantify male sperm movement we stained males with MitoTracker Red and mated them to wild-type hermaphrodites. Sperm from *sdha-2(tm1420)* males (n = 11 gonad arms) failed to migrate through the uterus, remaining largely near the vulva, while sperm from wild-type males (n = 11 gonad arms) migrated successfully to the spermatheca (Figures 5I and 5J). We divided the uterus into thirds and tracked the movement of sperm localized in Zone 2 of the uterus.^24^ Although a few *sdha-2(tm1420)* sperm were motile, their velocity was significantly less than wild-type sperm (Figure 5K). Together with the *in vitro* sperm activation assay results, these data suggest that *in vivo sdha-2(tm1420)* male sperm and hermaphrodite self-sperm both fail to activate and grow the pseudopod required for motility and are thus motility defective.

### Loss of other TCA cycle and electron transport chain components results in sperm defects

As a subunit of mitochondrial complex II, SDHA plays a critical role in both the TCA cycle and the ETC. Therefore, *sdha-2* mutant infertility may be due to problems with the TCA cycle and/or the ETC. We performed a candidate screen to investigate whether mutants in other components of the TCA cycle or ETC displayed a fertility defect similar to *sdha-2* mutants. We obtained strains mutant in the *sdha-2* homolog *sdha-1*, other subunits of mitochondrial complex II (*sdhb-1* and *sdhc-1*), *fum-1* which encodes an enzyme predicted to catalyze the reversible conversion of fumarate to malate in the TCA cycle, and *icl-1* which encodes an enzyme predicted to convert isocitrate and acetyl-CoA into succinate, malate, and CoA via a glyoxylate intermediate in the glyoxylate shunt.^25^ The glyoxylate shunt bypasses two intermediate TCA cycle steps^25^^,^^26^ (Figure 5D), and is conserved in bacteria, fungi, protists, nematodes, and plants, but absent in mammals.

*sdha-1*, *sdhb-1*, *sdhc-1* and *fum-1* are essential genes. RNAi knockdown and homozygous loss of function mutations in these genes result in sterility, embryonic lethality, and/or larval arrest,^27^^,^^28^^,^^29^^,^^30^^,^^31^^,^^32^^,^^33^ and so these mutant strains are maintained as heterozygotes by the use of genetic balancers. As it is not possible to perform accurate brood size assays on balancer strains, we counted the number of sperm per spermatheca and looked for sperm aberrantly localized throughout the uterus in heterozygote hermaphrodites, which might display a subtle reduction of function phenotype. *icl-1* and mutants carrying the *sdhc-1(kn1)* reduction of function allele can be maintained as homozygotes, so homozygous hermaphrodites were assayed.

*sdha-1(ok1908) +/−* animals had significantly less sperm per spermatheca than wild-type, displaying an intermediate phenotype between wild-type and *sdha-2(tm1420)* animals (Figures 5A and 5B). These results are consistent with a role for *sdha-1*, like *sdha-2*, in normal sperm activation—although it cannot be ruled out that *sdha-1* mutants have a different defect to *sdha-2*, such as fewer sperm.

*sdhb-1* and *fum-1* mutants showed no significant difference in numbers of sperm per spermatheca compared with wild-type (Figure 5A). However, since heterozygote animals were used, a mild effect may not have been detected in this assay and cannot be ruled out. *sdhc-1(ok909)* +/− animals displayed high variability in the number of sperm per spermatheca (Figure 5A). Although there was no significant difference compared with wild type, sperm could be observed throughout the uterus of some animals (Figure 5C), suggesting that *sdhc-1* may also contribute to normal sperm activation. Intriguingly, the *sdhc-1(kn1)* reduction of function allele had wild-type numbers of sperm per spermatheca (Figure 5A), despite previous studies recording decreased brood size.^34^^,^^35^ Given our results, it is possible that *sdhc-1(kn1)* mutants have a female germline or embryonic survival defect and an additional sperm defect that only becomes apparent in the more severe null allele.

Interestingly, *icl-1* mutants displayed an intermediate phenotype between wild-type and *sdha-2(tm1420)* animals (Figures 5A and 5B). To investigate *icl-1* fertility and potential interactions with *sdha-2* further, we performed a brood assay on *sdha-2(tm1420)*, *icl-1(ok531)*, and *sdha-2(tm1420); icl-1(ok531)* mutants, with wild-type and each mutant strain isolated from a single cross to ensure a homogeneous genetic background. *icl-1(ok531)* mutants again displayed an intermediate phenotype between wild-type and *sdha-2(tm1420)* animals with a significant decrease in live progeny numbers, but did not show an increase in unfertilized eggs (Figures 5E–5H). The *sdha-2(tm1420); icl-1(ok531)* double mutant displayed a severe reduction in live progeny and concurrent increase in unfertilized oocytes, which was comparable to the fertility of animals carrying the *sdha-2(tm1420)* mutation only (Figures 5E–5H). A shared mutant phenotype and the absence of an additive effect in the double mutant suggest that the two proteins may function in the same pathway to impact fertility. ICL-1 and SDHA-2 both function to process metabolites in the connected pathways of the glyoxylate shunt and TCA cycle respectively, and both mutants potentially have a reduction in malate. Thus, a metabolite imbalance may underlie the sperm defect.

### Impaired mitochondrial fission/fusion dynamics underlie sperm defects

**Figure 6:**
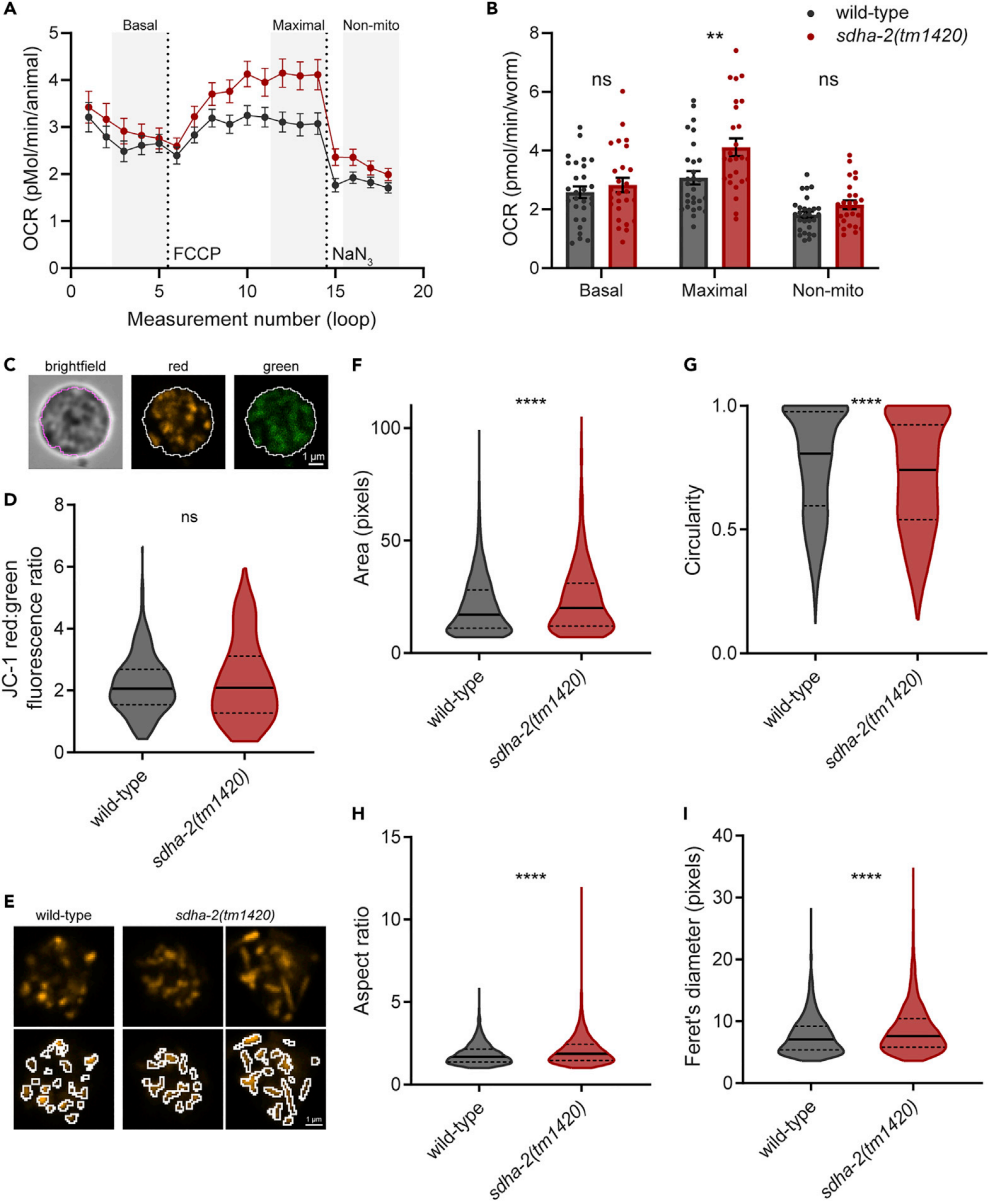
*sdha-2* mutant sperm display aberrant mitochondrial morphology (A and B) Oxygen consumption rate (OCR) in day 1 adults was assessed using a Seahorse Analyzer. OCR was measured under basal conditions (basal respiration), in response to the mitochondrial uncoupler carbonylcyanide-4-(trifluoromethoxy)-phenylhydrazone (FCCP) (maximal respiration), and the complex IV and V inhibitor sodium azide (NaN_3_) (non-mitochondrial OCR). (A) Mean OCR trace, with compound additions marked by dotted lines. (B) Summary data generated by calculating the means of the measurements from the final three loops of each condition. (A and B) Data are mean ± SEM; n = 26-28 pooled from three independent replicates. Replicate data is available in Figures S2A–S2C. Comparisons were performed using unpaired t-tests, ∗∗p ≤ 0.01. (C) Mitochondrial membrane potential (MMP) was assessed by staining isolated day 1 adult male spermatids with JC-1 dye. A generous binary threshold was generated using the green channel, which was then used to quantify green and red fluorescence intensity of spermatids. An outline of the threshold is displayed on a representative wild-type spermatid in magenta (brightfield) or white (red and green). (D) Ratio of red:green fluorescence intensity in JC-1-stained spermatids, proportional to MMP. Violin plots display the median (solid line) and quartiles (dashed lines); n = 421 spermatids for wild-type and 146 for *sdha-2(tm1420)* from >6 males. Comparison was performed using an unpaired t-test. (E) JC-1-stained spermatid mitochondria were detected in the red channel by an ImageJ pipeline and outlined in white here. Representative wild-type and *sdha-2(tm1420)* spermatids in the red channel are displayed, without and with ImageJ outline. Two *sdha-2(tm1420)* spermatids are displayed as examples of mild and severe mitochondrial morphology defects. (F–I) Following mitochondrial detection as in (E), mitochondrial morphology parameters in the red channel were calculated in ImageJ, including (F) area, (G) circularity (where 1 is a perfect circle), (H) aspect ratio (major axis/minor axis) and (I) Feret’s diameter (the longest distance between any two points in an object). Scale bar = 1 μm. Violin plots display the median (solid line) and quartiles (dashed lines); n = 4781 mitochondria for wild-type and 2564 for *sdha-2(tm1420)* from 4-6 males. Comparisons were performed using unpaired t-tests, ∗∗∗∗p ≤ 0.0001.

Given SDHA-2 is the catalytic subunit of complex II, we wondered whether ETC function was compromised in *sdha-2* mutants. Wild-type and *sdha-2(tm1420)* day one adult animals displayed no significant difference in basal oxygen consumption rate (OCR) using a Seahorse Analyser, indicating that mitochondrial respiration at the whole-animal level is unaffected by loss of SDHA-2 (Figures 6A, 6B, and S2A–S2C). Surprisingly, the addition of the mitochondrial uncoupler FCCP to assess maximal respiration revealed that *sdha-2(tm1420)* mutants displayed significantly increased maximal OCR compared with wild-type (Figures 6A, 6B, and S2A–S2C). We observed no difference in non-mitochondrial OCR, assessed by the addition of the complex IV and V inhibitor sodium azide.

Since we did not observe a decrease in mitochondrial respiration at the whole-animal level, we investigated ETC function specifically in sperm by assessing sperm mitochondrial membrane potential (MMP). The fluorescent dye JC-1 accumulates in mitochondria proportionally to MMP and as concentrations of JC-1 increase, fluorescence emission switches from green to red, and thus red:green ratio is a measure of MMP. We stained isolated spermatids with JC-1 and measured the red and green fluorescence intensity of whole spermatids (Figure 6C). We observed no significant difference in the red:green ratio between wild-type and *sdha-2(tm1420)* spermatids (Figure 6D), consistent with our findings at the whole-animal level. However, *sdha-2(tm1420)* spermatids displayed greater variation than wild-type, suggesting that spermatid MMP may be more variable in *sdha-2* mutants.

We quantified the number of mitochondria in the red (healthy mitochondria) and green (unhealthy mitochondria) channels in wild-type and *sdha-2* animals. *sdha-2* mutants and wild-type animals had comparable numbers of mitochondria in the red channel, but interestingly *sdha-2* mutants had significantly more mitochondria in the green channel indicating a higher proportion of mitochondria with low MMP (Figure S2D).

Staining mitochondria with JC-1 dye also enabled the assessment of mitochondrial morphology. Healthy (red fluorescing) mitochondria in wild-type spermatids were generally rounded or oval as expected.^36^ In contrast, some mitochondria in *sdha-2(tm1420)* spermatids appeared elongated, filamentous, or hyperfused (Figure 6E). We quantified these differences in individual mitochondria using an automated pipeline in ImageJ (see STAR methods) (Figure 6E). Mitochondria in *sdha-2(tm1420)* spermatids were significantly larger and less circular than wild-type mitochondria (Figures 6F and 6G). Furthermore, *sdha-2(tm1420)* mitochondria’s aspect ratio and Feret’s diameter were significantly greater than wild-type (Figures 6H and 6I). Mitochondria in the green channel displayed the same alterations to morphology metrics (Figures S2E–S2H). Healthy mitochondria undergo continuous fission and fusion, and so the presence of some elongated mitochondria is normal. However, the presence of many filamentous mitochondria and some hyperfused mitochondria in *sdha-2(tm1420)* spermatids and not in wild-type suggests that loss of SDHA-2 results in aberrant mitochondrial fission/fusion dynamics.^37^ The relationship between mitochondrial fission/fusion dynamics and health is complex, but generally elevated fusion and/or inhibited fission is a response to mitochondrial stress.^38^

### The mitochondrial unfolded protein response is not elevated in whole *sdha-2* mutant animals

Since *sdha-2* mutants displayed aberrant mitochondrial morphology and dynamics, we investigated the mitochondrial unfolded protein response (UPR_mt_)—a conserved response to mitochondrial stress.^33^^,^^39^ UPR_mt_ has been shown to be induced by the knockdown of several ETC proteins in *C. elegans*, including other SDH components.^33^^,^^40^^,^^41^^,^^42^ We quantified fluorescence in *sdha-2* and wild-type L4 and Day 1 adult animals expressing either the *hsp-6::gfp* or *hsp-60::gfp* integrated transcriptional reporters.^33^

**Figure 7:**
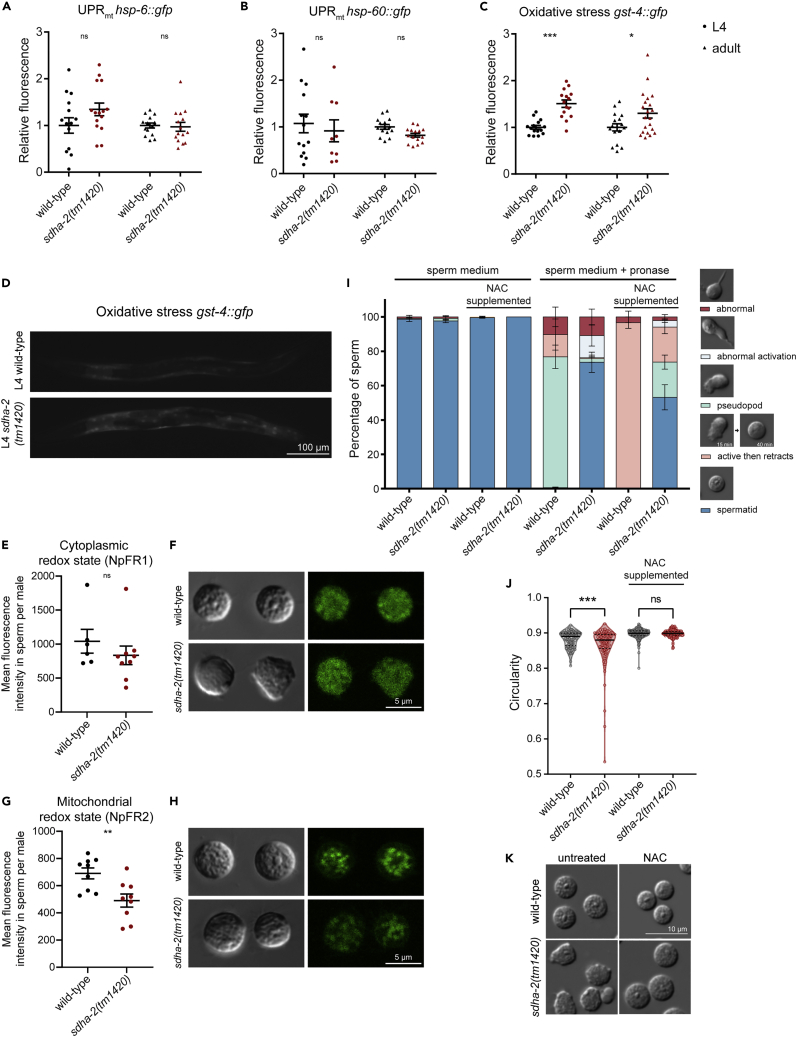
Loss of SDHA-2 does not induce mitochondrial unfolded protein response, but does induce redox imbalance (A and B) Mitochondrial unfolded protein response was assessed using the fluorescent transcriptional reporters (A) *hsp-6::gfp* and (B) *hsp-60::gfp*. (C) Oxidative stress was assessed using the fluorescent transcriptional reporter *gst-4::gfp*. (A–C) Data are mean ± SEM; n = 9-21. Comparisons are between wild-type and *sdha-2* at the indicated age, and were performed using two-way ANOVA with Sidak’s post hoc test, ∗p ≤ 0.05, ∗∗p ≤ 0.01, ∗∗∗p ≤ 0.001. (D) Representative fluorescent images of L4 animals of the indicated genotype. (E–H) Redox status in sperm was assessed by incubating isolated day 1 adult male spermatids with the fluorescent redox probes (E and F) NpFR1 (cytoplasmic) and (G and H) NpFR2 (mitochondrial). (E and G) Data are mean ± SEM; n = 6-9 males from two independent replicates. Comparisons were performed using unpaired t-tests, ∗∗p ≤ 0.01. (F and H) Representative wild-type and *sdha-2(tm1420)* spermatids incubated with NpFR1 or NpFR2. Scale bar = 5 μm. (I) *In vitro* sperm activation assay. Virgin adult males grown on unsupplemented (NGM) or NAC-supplemented plates were dissected in sperm medium with or without Pronase, as indicated. Data are mean ± SEM. n ≥ 180 sperm from ≥5 males. (J) Spermatid morphology upon NAC supplementation with (K) representative images. Scale bar = 10 μm. Circularity was quantified using ImageJ, where 1 is a perfect circle. n = 115-210 spermatids from 3 males. Comparisons were performed using one-way ANOVA with Tukey’s post hoc test, ∗∗∗p ≤ 0.001.

We quantified relative fluorescence intensity in whole animals and found no significant difference between wild-type and *sdha-2(tm1420)* mutants in either of the two UPR_mt_ reporters (Figures 7A and 7B). These results suggest that UPR_mt_ is not upregulated in *sdha-2* mutants at the whole animal level. As UPR_mt_ is mediated by the transcription factor ATFS-1 which is regulated by MMP,^40^^,^^43^ our results are consistent with our finding that MMP is unchanged in *sdha-2* mutants.

### Loss of SDHA-2 function results in redox imbalance

Inhibition of SDH can result in elevated reactive oxygen species (ROS) levels under some conditions.^44^ An imbalance between ROS production and antioxidant mechanisms results in oxidative stress. Mammalian spermatozoa are particularly sensitive to oxidative stress as they lack the cytoplasmic enzyme repair systems of other tissues, and so are incapable of repairing oxidative stress-induced damage.^45^ Elevated ROS levels are cytotoxic, resulting in decreased sperm motility, DNA integrity, and capacity to fertilize oocytes.^45^ Exposure to paraquat generates mitochondrial ROS and induces oxidative stress in *C. elegans*, and paraquat-treated males display impaired sperm activation.^46^ To determine whether loss of *sdha-2* results in oxidative stress in *C. elegans*, we used a *gst-4::gfp* transcriptional reporter of oxidative stress.^47^ We quantified whole-animal relative fluorescence intensity and found that *sdha-2(tm1420)* mutants displayed a small but significant increase in fluorescence compared with wild-type animals at both L4 and day 1 of adulthood (Figures 7C and 7D). The difference was marginally greater in L4 animals when sperm are being made. This suggests that loss of SDHA-2 function induces oxidative stress at the whole animal level which may contribute to defective sperm activation and motility.

To examine the redox state of sperm, we stained sperm with the fluorescent redox probes NpFR1 (cytoplasmic) and NpFR2 (mitochondrial).^48^^,^^49^ These probes are almost non-fluorescent in the reduced state and fluorescent when oxidized, and so fluorescence intensity is a measure of the relative redox state. *sdha-2* mutant sperm displayed no significant differences compared to wild type in the cytoplasm (Figures 7E and 7F), but significantly decreased fluorescence intensity compared with wild-type in mitochondria (Figures 7G and 7H), suggesting that loss of SDHA-2 disrupts sub-cellular redox balances in sperm. It is unclear why *sdha-2* mutants display increased oxidative stress at the whole animal level but decreased oxidative capacity in sperm, and this warrants further investigation.

Since we observed changes in oxidation levels in *sdha-2* mutants, we wondered whether antioxidant supplementation could rescue the fertility defect. Dietary supplementation with N-acetylcysteine (NAC) from birth through to reproductive maturity caused an increase in the percentage of activated sperm in *sdha-2* mutant males (Figure 7I), although it did not fully rescue to wild-type levels. NAC supplementation also caused an increase in sperm that activated and then retracted their pseudopod over a 40-min time course in both the wild-type and *sdha-2* mutant males (7I). NAC supplementation also rescued the spermatid morphology defect observed in sdha-2 mutant sperm, increasing the spermatid circularity up to wild-type levels (Figures 7J and 7K).

### Sporadic, non-heritable reversion to normal fertility implicates mitohormesis

**Figure 8:**
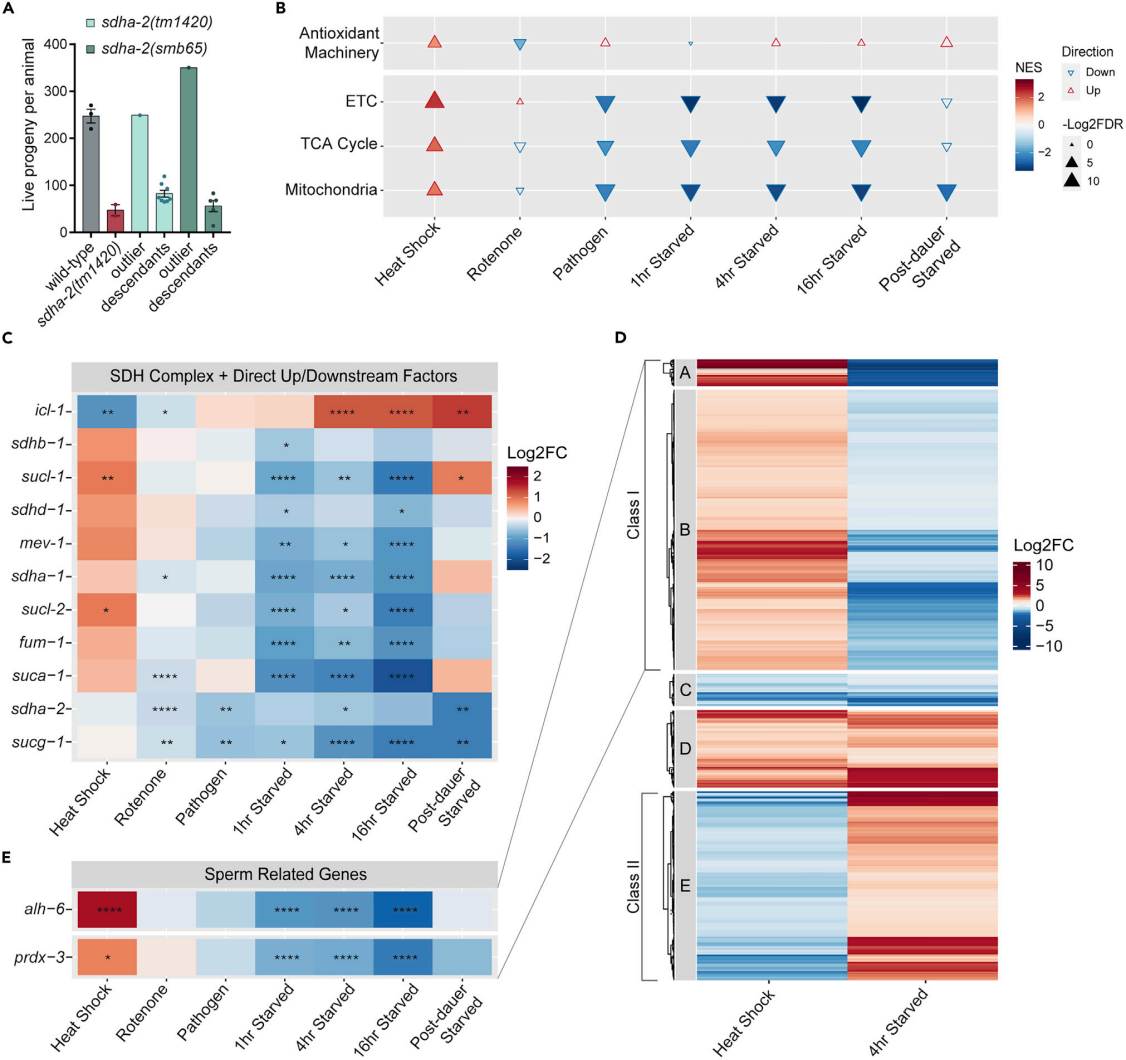
*sdha-2* mutants display rare, spontaneous, non-heritable reversion to wild-type fertility (A) Rarely, a *sdha-2* mutant animal displayed a large outlier brood size. We screened descendants of these outliers in replicate (n = 5-8, 2-3 for controls) and could not recapitulate the large brood size. Data are mean ± SEM where applicable. Descendant and control data were collected in one assay, outlier data was collected in separate, previous assays. (B) Gene set enrichment analysis (GSEA) of mRNA transcripts after each stress exposure. A positive normalized enrichment score (NES) indicates that the gene set was upregulated after stress exposure. Hollow triangles indicate a lack of statistical significance (FDR>0.05, -Log2(FDR) < 4.3). Gene sets were based on GO term categories and manually curated for greater accuracy, see STAR Methods for details. (C) Heatmap of SDH complex members and immediate up and downstream pathway factors. (D) Heatmap of all genes significantly differentially expressed in both heat shock and 4-h starvation datasets. Clustering was performed using Euclidean row distance and ward.D2 clustering method. (E) Heatmap of known sperm-related genes found within the Class I gene set.

Whilst performing brood size assays, we noticed that very rarely a *sdha-2* mutant control animal produced a brood indistinguishable from wild-type in live progeny and unfertilized oocyte numbers. We collected descendants from these parents, confirmed the *sdha-2* mutant genotype, and repeated brood size assays (Figure 8A). We could not recapitulate the large brood size originally observed for any line indicating that, in rare instances, *sdha-2* mutant individuals display reversion to wild-type brood size, and that this reversion is not heritable. Lack of inheritance suggests that the underlying cause is non-genetic, and not due to spontaneous suppressor mutations. We observed this reversion in both *sdha-2(tm1420)* and AKA36/*sdha-2(smb65)* animals, indicating that reversion is not allele specific. Over the course of this study, we have assayed the brood size of 162 *sdha-2* mutant animals and observed reversion in 7, and therefore estimate the reversion frequency at 4%. Given this infrequency and that the phenotype is not heritable, pursuing the mechanism behind reversion presents a challenge.

Mild environmental stress can induce subtle mitochondrial stress which paradoxically results in enhanced stress defense and physiological benefits, in a process called mitohormesis.^50^^,^^51^ Despite the standardized conditions under which our animals were grown, perhaps a variable environmental factor resulted in the reverted animals experiencing a mild stress during development, causing mitochondrial modulation which resulted in improved fertility. This improvement could have involved the direct replacement of SDHA-2 activity by the upregulation of *sdha-1*, or changes in expression of other mitochondrial components which could compensate for molecular deficiencies or imbalances in the absence of SDHA-2 to restore sperm function. Alternatively, the improvement could have been caused by an increased ability to cope with damage resulting from loss of SDHA-2, perhaps by the upregulation of antioxidant machinery.

We examined RNAseq data from 12 environmental stress conditions from 5 different published datasets (see STAR Methods) (Figure S3) to see whether such stresses modulate the expression of 1) the SDH complex and immediate up and downstream pathway factors, 2) TCA cycle genes, 3) ETC genes, 4) mitochondrial genes more broadly, 5) antioxidant pathway genes. Initial gene set enrichment analysis (GSEA) revealed that both pathogen exposure and starvation in adulthood significantly downregulated ETC, TCA cycle, and mitochondrial gene sets, but not antioxidant pathway genes (Figure 8B). Re-fed adults that underwent starvation-induced dauer arrest also showed a general trend of down-regulation. Conversely, mild heat shock in N2 animals significantly upregulated all tested gene sets. Exposure to the ETC complex 1 inhibitor rotenone, a mild oxidative stressor, significantly downregulated antioxidant activity and resulted in no other significant changes (Figure 8B). At the individual gene level, we found that the majority of the SDH complex and the immediate up and downstream factors (although not SDHA-2) were differentially expressed in the same direction as seen in the GSEA (Figures 8B and 8C). Strikingly, *icl-1*, which may work in the same pathway as *sdha-2* to impact sperm health (Figure 4), showed the opposite expression pattern to other SDH complex genes (Figure 8C). Loss of function of other SDH complex members is associated with increased *icl-1* activity,^52^^,^^53^^,^^54^ and we consistently found that SDH genes and *icl-1* displayed opposite expression changes following stress exposure. Potentially, upregulation of *icl-1* after certain environmental stresses may activate the glyoxylate shunt and bypass complex II, ameliorating deleterious effects from loss of SDHA-2. These analyses confirm that the SDH complex and intersecting pathways are indeed stress responsive, and could be modulated in our observed revertants.

Starvation and heat shock showed opposing patterns in our ETC and TCA cycle groups for the GSEA (Figure 8B) and also in the individual SDH complex genes analysis (Figure 8C). If this pattern holds true on a larger scale it could clarify our understanding of the reversion effect. To investigate, we performed hierarchical clustering on all the genes significantly differentially expressed in both heat shock and 4hr starvation (the midpoint of the starvation time course) (Figure 8D). These 657 genes are separated into 5 distinct groups. Groups A (29 genes) and B (304 genes) were upregulated in heat and downregulated in starvation, matching the overall pattern observed in the GSEA. We named this combined group Class I. Group E showed the opposite pattern and matched the *icl-1* expression pattern, and we named these genes Class II. Groups C and D were consistently down or upregulated, respectively, in both stress conditions. KEGG pathway analysis of the Class I genes found 27 significantly enriched pathways including “TCA Cycle” as expected and many other metabolic pathways, as well as other relevant terms including antioxidant pathways such as “Glutathione metabolism” (Figure S4). KEGG analysis of the Class II genes found only 3 significantly enriched terms: “FoxO signaling pathway” and two autophagy pathways. In mice, FoxO is known to be expressed during late spermatogenesis,^55^ and the pathway is crucial to proper spermatogenesis and sperm maturation,^56^ suggesting a role for this pathway in fertility reversion. No pathways were significantly enriched for the C or D groups.

Since we saw a mild increase in oxidative stress in *sdha-2* mutants, we hypothesized that revertants may have increased antioxidant pathway function. We found that in multiple stress conditions, many genes involved in the *C. elegans* antioxidant pathways (GO:0016209) were significantly differentially expressed (Figure S5), although they did not show a clear pattern of up or downregulation. When separated into descendant GO term categories, peroxiredoxin genes (GO:0051920) showed a similar pattern to the SDH complex genes Figure S5), and *prdx-3* was identified within our Class I gene set (Figures 8D and 8E). Repression of peroxiredoxin activity causes a significant decrease in brood size in *C. elegans*,^57^ and causes significantly decreased sperm motility and male fertility in mammals.^58^ Another antioxidant gene, *sod-2*, also displayed the same pattern of significant downregulation in starvation, with a visible but not significant upregulation after heat shock (Figure S5). *sod-2* is thought to be the only mitochondrially active superoxide dismutase (SOD) in *C. elegans* adults^59^ and is known to be crucial in *C. elegans* sperm activation.^60^ Thus, antioxidant pathways are stress responsive, and stress-induced upregulation of these antioxidant genes could be related to the occasional fertility reversion in *sdha-2* mutant animals.

Therefore, we conclude that stress has striking effects on the expression of factors intimately connected to SDHA-2, and the fertile revertants could have experienced a mild stress during development that resulted in mitohormesis and single-generation changes to mitochondrial dynamics and/or antioxidant pathways which alleviated the *sdha-2* deficiency-dependent infertility.

## Discussion

**Figure 9:**
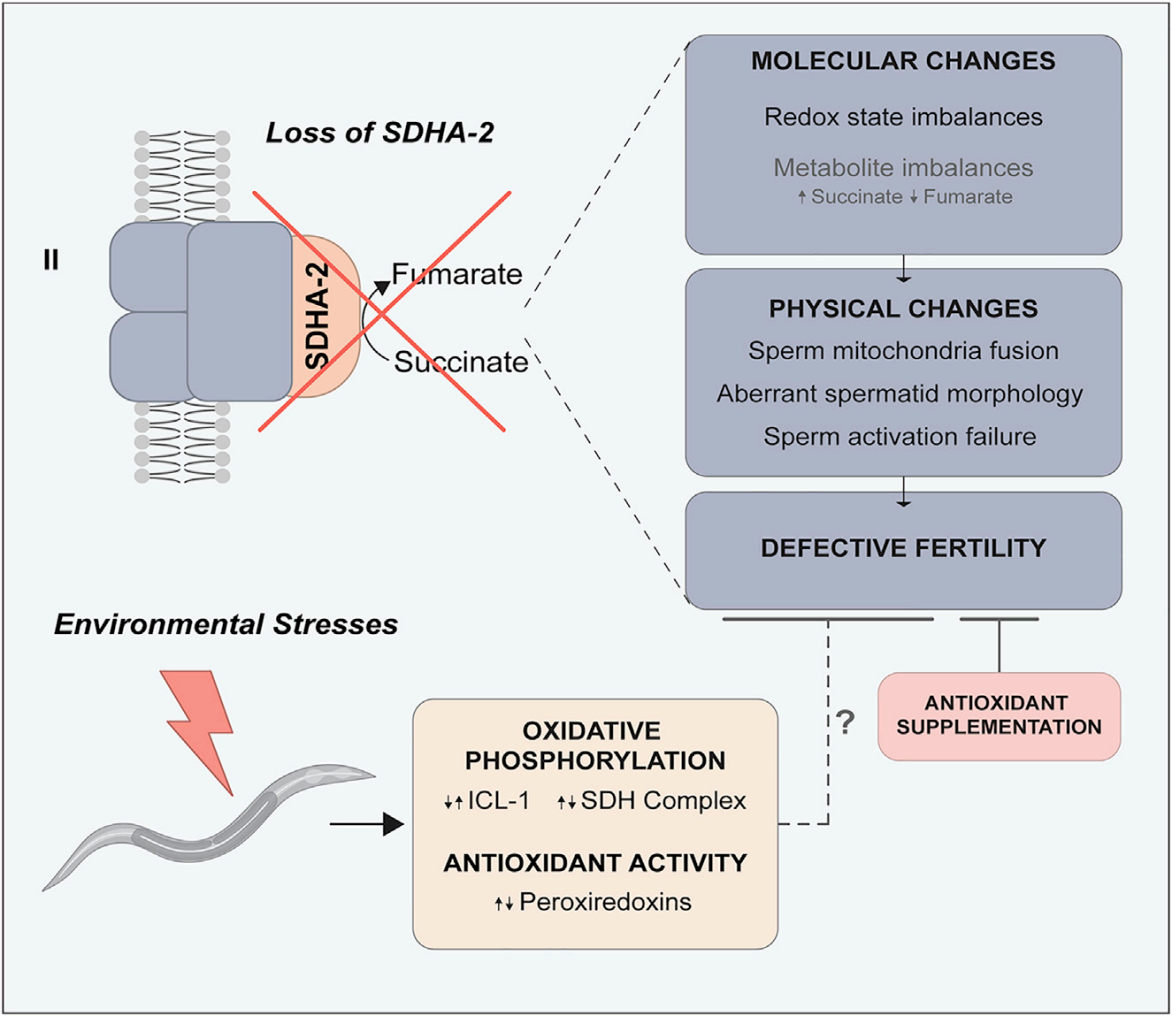
Model of the effect of SDHA-2 loss on male fertility and the potential interaction with environmental stresses

Sperm motility is essential for successful reproduction. Here we demonstrate that the mitochondrial enzyme SDHA-2 is required for sperm activation and motility, and thus male fertility. *sdha-2* mutant sperm are misshapen, and harbor mitochondria with morphological abnormalities including an increased prevalence of elongated mitochondria, indicating disrupted fission/fusion dynamics and mitochondrial stress. These findings highlight the critical role of SDH and mitochondria function in sperm health (Figure 9).

Our data provide insight into the role of SDH function in sperm health at the molecular level, examining the contributions of oxidative phosphorylation disruption, metabolite imbalance, and oxidative stress. As a key contributor to the ETC, loss of SDHA-2 is predicted to disrupt oxidative phosphorylation, which may cause decreased ATP production in sperm. Indeed, RNAi knockdown of *sdha-2* results in decreased ATP levels in whole animals.^61^ However, we found no difference in oxygen consumption at the whole animal level and no significant decrease in mean mitochondrial membrane potential (MMP) in *sdha-2* mutant sperm suggesting that *sdha-2* sperm dysfunction is not due to decreased oxidative phorphorylation. It is worth noting that SDHA-1 and -2 may have a redundant function or specific expression profiles, which could explain the lack of *sdha-2* mutant dysfunction observed in these metrics.

Metabolite imbalance likely contributes to SDHA-2-dependent sperm defects. We have shown that *icl-1* mutants, like *sdha-2* mutants, have fewer sperm in the spermatheca, implicating an imbalance in TCA cycle metabolites in this phenotype. Loss of ICL-1 also results in a reduced brood size, albeit to a lesser extent than the loss of SDHA-2. Loss of ICL-1 and SDHA-2 could result in a decreased concentration of downstream TCA cycle metabolites including malate, decreased TCA cycle products such as fatty acids and signaling molecules, or toxic accumulation of upstream metabolites. A bottleneck-effect due to the absence of SDHA-2 could cause increased abundance of upstream succinyl-CoA. Succinyl-CoA is the putative cofactor of lysine succinylation, a post-translational protein modification of emerging significance. Indeed, SDH inhibition results in increased succinyl-CoA and hyper-succinylation in mammalian cells,^62^ and loss of succinyl-CoA ligase, the enzyme immediately preceding SDH in the TCA cycle, results in global protein hyper-succinylation in yeast, fish and mammals.^63^^,^^64^ Hyper-succinylation of mitochondrial proteins is associated with inhibited protein and enzyme function and mitochondrial dysfunction,^62^^,^^63^^,^^65^ providing an intriguing hypothesis that loss of SDHA-2 may cause sperm dysfunction due to hyper-succinylation.

Furthermore, we have shown that oxidative stress is induced in *sdha-2* mutants (Figures 7C and 7D). This is likely a result of either an increase in the production of ROS or decrease in TCA cycle intermediates which can act as antioxidants,^66^^,^^67^ due to the disruption of the ETC and/or TCA cycle in the absence of SDHA-2. Oxidative stress is known to have deleterious effects on sperm health including impaired sperm motility,^45^^,^^46^ and as the defect is partially rescued by NAC supplementation (Figures 7I–7K), SDHA-2-associated oxidative stress may have a causal role in sperm dysfunction.

Interestingly, the sperm defects that we observe in *sdha-2* mutants are reminiscent (but much stronger than) defects observed in the proline catabolism gene *alh-6* and the FAD synthetase gene *flad-1*.^36^^,^^46^ Loss of *alh-6* results in the accumulation of toxic proline catabolism intermediates and impaired ROS homeostasis, contributing to sperm dysfunction, which can be rescued by dietary supplementation with the FAD precursor riboflavin.^36^^,^^46^ *C. elegans* SDHA-2 is predicted to use FAD as a cofactor in the conversion of succinate to fumarate.^68^^,^^69^ The reduction in FAD levels following the loss of *alh-6* and *flad-1*^36^ may result in decreased availability of FAD as a cofactor for SDHA-2, thereby resulting in decreased SDHA-2 function. We identified *alh-6* within our Class I gene set (Figures 8D and 8E), suggesting that *alh-6* and *sdha-2* may act in the same pathway. Thus, decreased SDHA-2 function could contribute to the milder sperm defects described by Yen and colleagues,^36^^,^^46^ while complete loss of SDHA-2 function described in our study results in dramatic sperm function failure. Preliminary analysis using RNAi knockdown of *alh-6* suggested that the reduction of ALH-6 causes dysregulated oogenesis unrelated to sperm defects, but strong conclusions about male fertility were unable to be drawn, potentially due to reduced RNAi efficacy in sperm, and this should be investigated further (Figure S6).

Finally, we uncovered instances of spontaneous reversion of SDHA-2 deficiency-dependent infertility. Our RNAseq analysis identified multiple pathways by which this rescue could be mediated, including the modulation of SDH components, ETC and TCA cycle factors, and antioxidant pathways. This spontaneous reversion indicates that the *sdha-2* infertility defect is rescuable and provides a unique and exciting opportunity to uncover potential therapeutics for SDH deficiency-associated pathologies.

This study has revealed a new role for SDHA in *C. elegans* sperm activation and motility. Hermaphrodite and male sperm have distinct sperm activation pathways, with distinct genetic requirements.^70^ We have shown that SDHA-2 is important for both hermaphrodite and male sperm function, uncovering a fundamental requirement in sperm activation that spans sex-specific pathways. Our results implicate the ETC and TCA cycle in this process and provide future directions to further investigate the potential involvement of metabolite and ROS homeostasis. Fully characterizing the *sdha*-*2* mutant sperm dysfunction and spontaneous rescue is expected to provide therapeutic pathways to treating human SDH deficiency-associated infertility and other pathologies.

### Limitations of the study

In *C. elegans*, subunit A of the mitochondrial SDH complex is encoded by two homologs, *sdha-1* and *sdha-2*, likely derived from a recent gene duplication event.^71^ *sdha-1* is an essential gene, and homozygous mutants are non-viable. In contrast, *sdha-2* mutants are viable. It will be important to determine whether the differences in phenotype severity between the homologs are due to expression pattern (for example, is *sdha-2* expression restricted to sperm and their progenitors), and/or whether SDHA-1 might also have a role in sperm activation and motility. It will also be important to confirm whether *sdha-2* mutants have any additional defects in spermatogenesis.

## Data and materials availability

All data needed to evaluate the conclusions in the article are present in the article and/or the Supplementary Materials.

## STAR★Methods

### Key resources table

**Table:** 

REAGENT or RESOURCE	SOURCE	IDENTIFIER
**Bacterial and virus strains**		

*E. coli* OP50	Caenorhabditis Genetics Center (CGC)	N/A
*E. coli* HT115(DE3) with *alh-6* and empty RNAi clones	N/A	N/A

**Chemicals, peptides, and recombinant proteins**		

Pronase	Sigma	Cat#P5147
Monensin	Sigma	Cat#M5273
Mitotracker	Thermofisher	Cat#M7512
JC-1	Invitrogen	Cat#T3168
NpFR1	Yeow et al.^49^	N/A
NpFR2	Kaur et al.^48^	N/A
N-acetyl Cysteine	Sigma	Cat#A7250

**Critical commercial assays**		

Dneasy blood and tissue kit	QIAGEN	Cat#69504
TruSeq Nano DNA Sample Preparation Kit	Illumina	Cat#20015965

**Experimental models: Organisms/strains**		

*C. elegans*: N2: Wild-type, Bristol isolate	CGC	N/A
*C. elegans*: SX461: *mjIs31[ppie-1::gfp::h2b]* II	(Ashe et al., 2012)	N/A
*C. elegans*: AKA36^1^: *set-32(ok1457)* I; *sdha-2(smb65)* I; *mjIs31[ppie-1::gfp::h2b]* II	Woodhouse et al.^20^	N/A
*C. elegans*: FX01420 (used in Figure 1B): *sdha-2(tm1420)* I	NBRP	N/A
*C. elegans*: FX31204: *col-60(tm10278)* I	NBRP	N/A
*C. elegans*: RB1480: *nra-2(ok1731)* I	International *C. elegans* Gene Knockout Consortium	N/A
*C. elegans*: EU1135: *tba-1(or346)* I	(Phillips et al., 2004)	N/A
*C. elegans*: EU1161: *tba-1(or594)* I	(O’Rourke et al., 2011)	N/A
*C. elegans*: RB1185: *tba-1(ok1135)* I	International *C. elegans* Gene Knockout Consortium	N/A
*C. elegans*: FX31000: *mgl-2(tm8806)* I	NBRP	N/A
*C. elegans*: FX00355: *mgl-2(tm355)* I	NBRP	N/A
*C. elegans*: CB698: *vab-10(e698)* I	(Hodgkin, 1983)	N/A
*C. elegans*: VC117: *vab-10(gk45)* I	International *C. elegans* Gene Knockout Consortium	N/A
*C. elegans*: AKA156: *F30A10.15(smb55)* I; *mjIs31[ppie-1::gfp::h2b]* II	This paper. Outcrossed 2x following generation by CRISPR-Cas9	N/A
*C. elegans*: AKA157: *marc-3(smb53)* I; *mjIs31[ppie-1::gfp::h2b]* II	This paper. Outcrossed 2x following generation by CRISPR-Cas9	N/A
*C. elegans*: AKA136 (used in every instance except Figure 1B): *sdha-2(tm1420)* I; *mjIs31[ppie-1::gfp::h2b]* II	This paper. Outcrossed 7x from FX01420	N/A
*C. elegans*: AKA145 (referred to as AKA36[*sdha-2(+)*] in this paper): *set-32(ok1457)* I; *mjIs31[ppie-1::gfp::h2b]* II	This paper. Derived from AKA36: *sdha-2(smb65)* repaired back to wild-type sequence by CRISPR-Cas9. Outcrossed 4x	N/A
*C. elegans*: AKA199: *sdha-2(smb65)* I; *mjIs31[ppie-1::gfp::h2b]* II	This paper. Outcrossed 6x following generation by CRISPR-Cas9	N/A
*C. elegans*: AKA59: *mjIs31[ppie-1::gfp::h2b]* II; *rhIs4[glr-1::gfp]* III; *him-8(e1489)* IV	Woodhouse et al.^20^	N/A
*C. elegans*: AKA60: *set-32(ok1457)* I; *sdha-2(smb65)* I; *mjIs31[ppie-1::gfp::h2b]* II, *rhIs4[glr-1::gfp]* III; *him-8(e1489)* IV	Woodhouse et al.^20^)	N/A
*C. elegans*: AKA205: *sdha-2(tm1420)* I; *mjIs31[ppie-1::gfp::h2b]* II, *rhIs4[glr-1::gfp]* III; *him-8(e1489)* IV	This paper	N/A
*C. elegans*: AKA207: *set-32(ok1457)* I; *mjIs31[ppie-1::gfp::h2b]* II, *rhIs4[glr-1::gfp]* III; *him-8(e1489)* IV	This paper	N/A
*C. elegans*: AKA25: *mjIs31[ppie-1::gfp::h2b]* II; *him-8(e1489)* IV	This paper. Outcrossed 1x from CB1489	N/A
*C. elegans*: AKA55: *set-32(ok1457)* I; *sdha-2(smb65)* I; *mjIs31[ppie-1::gfp::h2b]* II; *him-8(e1489)* IV	This paper	N/A
*C. elegans*: AKA131: *sdha-2(tm1420)* I; *him-8(e1489)* IV	This paper	N/A
*C. elegans*: VC1434: *+/szT1 [lon-2(e678)]* I; *sdha-1(ok1908)/szT1* X, VC294 *sdhb-1(gk165)/mIn1 [mIs14 dpy-10(e128)]* II	International *C. elegans* Gene Knockout Consortium	N/A
*C. elegans*: VC848: *sdhc-1(ok909)* III/*hT2 [bli-4(e937) let-?(q782) qIs48]* (I;III)^2^	International *C. elegans* Gene Knockout Consortium	N/A
*C. elegans*: TK22: *sdhc-1(kn1)* III^2^	Ishii et al.^35^	N/A
*C. elegans*: VC1497: *fum-1(ok1998)* III/*hT2 [bli-4(e937) let-?(q782) qIs48]* (I;III)	International *C. elegans* Gene Knockout Consortium	N/A
*C. elegans*: RB766: *icl-1(ok531)* V	International *C. elegans* Gene Knockout Consortium	N/A
*C. elegans*: ZB5206: *sdha-2(tm1420) I; icl-1(ok531)* V	This paper, outcrossed 4x	N/A
*C. elegans*: CB1489: *him-8(e1489)* IV	(Hodgkin et al., 1979)	N/A
*C. elegans*: SJ4100: *zcIs13[phsp-6::GFP]* V	Yoneda et al.^33^	N/A
*C. elegans*: AKA161: *sdha-2(tm1420)* I; *zcIs13[phsp-6::GFP]* V	This paper	N/A
*C. elegans*: SJ4058: *zcIs9 [phsp-60::GFP + lin-15(+)]* V	Yoneda et al.^33^	N/A
*C. elegans*: AKA164: *sdha-2(tm1420)* I; *zcIs9 [phsp-60::GFP + lin-15(+)]* V	This paper	N/A
*C. elegans*: CL2166: *dvIs19 [(pAF15)pgst-4::GFP::NLS]* III	Link and Johnson,^47^	N/A
*C. elegans*: AKA155: *sdha-2(tm1420)* I; *dvIs19 [(pAF15)pgst-4::GFP::NLS]* III	This paper	N/A

**Oligonucleotides**		

Sequences of CRISPR guides and repair templates used in this study	This study	Table S2
Alt-R® CRISPR-Cas9 tracrRNA	IDT	Cat#1072532

**Recombinant DNA**		

pHO4d-Cas9	Fu et al.^72^	Addgene plasmid #67881

**Software and algorithms**		

Custom Alt-R® CRISPR-Cas9 guide RNA design tool	IDT	https://sg.idtdna.com/site/order/designtool/index/CRISPR_CUSTOM
I-TASSER	Roy et al.^21^	https://zhanglab.ccmb.med.umich.edu/I-TASSER/
PyMOL	PyMOL by Schrödinger	https://pymol.org/
Fiji (ImageJ)	ImageJ	https://imagej.net/software/fiji/
GraphPad Prism 9	GraphPad	https://www.graphpad.com/ RRID: SCR_002798
CLC Genomics	QIAGEN	https://digitalinsights.qiagen.com/
WormBase	WormBase	https://wormbase.org/
R version 4.1.0	R Project	https://www.r-project.org/
RStudio version 1.4.1717	RStudio	https://www.rstudio.com/
Cutadapt version 1.8.3	Martin,^73^	https://doi.org/10.14806/ej.17.1.200
STAR aligner	Dobin et al.^74^	https://www.ncbi.nlm.nih.gov/pmc/articles/PMC3530905/
DESeq2	R Package (Love et al.^75^)	https://bioconductor.org/packages/release/bioc/html/DESeq2.html
GSEA	Broad Institute, Subramanian et al.^76^	https://www.gsea-msigdb.org/gsea/index.jsp
ggplot2	R library	https://ggplot2.tidyverse.org/
ComplexHeatmap	R Package Gu et al.^77^	https://bioconductor.org/packages/release/bioc/html/ComplexHeatmap.html
ShinyGO	Ge et al.^78^	http://bioinformatics.sdstate.edu/go/

### Resource availability

#### Lead contact

Further information and requests for resources and reagents should be directed to and will be fulfilled by the lead contact, Alyson Ashe (alyson.ashe@sydney.edu.au).

#### Materials availability

New *C. elegans* strains generated in this study will be made available by the lead contact upon request.

### Experimental model and subject details

*C. elegans* were cultured according to standard procedures.^79^ Animals were grown on Nematode Growth Medium (NGM) (2% (w/v) agar, 50 mM NaCl, 0.25% (w/v) peptone, 1 mM CaCl_2_, 5 μg/mL cholesterol, 25 mM K_3_PO_4_ and 1 mM MgSO_4_) seeded with *E. coli* strain OP50. In the NAC supplementation assay, NAC was added to the NGM plate mix to a final concentration of 5mM. Experiments were performed at 20°C. *C. elegans* strains used are listed in the key resources table. In all experiments except the UPR_mt_ and oxidative stress reporter assays, strain SX461 was used as the wild-type, which is N2 carrying an integrated nuclear germline GFP transgene *mjIs31[ppie-1::gfp::h2b]*. The transgene has no effect on development or health, and other strains in these experiments were in the same *mjIs31[ppie-1::gfp::h2b]* background. In the JC-1, UPR_mt_, oxidative stress reporter, and redox probe assays no strains carried *mjIs31[ppie-1::gfp::h2b]* to avoid interference with the fluorescent reporters. Note that all strains from which males were isolated (for brood size, sperm morphology, sperm activation, and JC-1 assays) also carried the *him-8(e1489)* mutation, which increases the prevalence of males in the population for ease of assay set up without altering the quality of sperm.

Note that the AKA36 strain used in Woodhouse et al.^20^ was outcrossed 6x from VC967; originally characterised as *set-32(ok1457)* I. In Figure 1 and Table S1 we characterise 9 additional non-synonymous background mutations on Chromosome I, including one in *sdha-2* which we named *smb65*. ‘AKA36’ is used throughout this study to refer to this combination of 10 mutations.

### Method details

#### Whole genome sequencing

Genomic DNA was purified from AKA36 and SX461 mixed population animals with Dneasy blood and tissue kit (QIAGEN). The protocol was modified with the addition of 40 μg RNAse after buffer ATL and proteinase K lysis, digested for 1 hour at 37°C. Libraries were prepared from genomic DNA using a TruSeq Nano DNA Sample Preparation Kit (Illumina). Libraries were whole-genome sequenced on the Illumina HiSeq 2500 platform with 100 bp paired end sequencing by the Australian Genome Research Facility (AGRF).

#### CRISPR/Cas9

All projects used CRISPR-Cas9 crRNA and tracrRNA and Cas9 nuclease as a ribonucleoprotein complex, and a single stranded oligonucleotide repair template. crRNAs, tracrRNAs and repair templates (Ultramer® DNA Oligos) were ordered from IDT. Cas9 nuclease included a C-terminal SV40-NLS and 6xHis tag, and was expressed from the plasmid pHO4d-Cas9 (Addgene plasmid #67881, a gift from Michael Nonet^72^), and produced by Protein Production and Characterisation at Sydney Analytical (University of Sydney).

crRNAs were designed using the IDT Custom Alt-R® CRISPR-Cas9 guide RNA design tool https://sg.idtdna.com/site/order/designtool/index/CRISPR_CUSTOM. crRNAs were selected based on proximity to the edit site, presence of 3′ GG(NGG)^80^ or G(NGG), and IDT on-target and off-target score.

Single stranded oligonucleotide repair templates contained 30–50 bp homology arms.^81^ Edits were 5′ of the PAM so the protospacer strand was used for repair.^82^ Each repair template incorporated the desired edit, a silent mutation at the PAM site where possible or alternatively 4–5 silent mutations in the guide sequence to prevent cleavage of the repair template, and silent mutation(s) to introduce a restriction enzyme cleavage site for ease of identifying edited animals.

We used a *dpy-10* co-CRISPR strategy for all projects, where *dpy-10* crRNA and repair template are included in the injection mix.^83^ Successful editing in offspring causes visible dumpy (dpy) (homozygous mutation) or roller (rol) (heterozygous mutation) phenotypes.

Injection mixes were adapted from the Dernburg lab (IDT online protocol, https://sg.idtdna.com/pages/education/decoded/article/genome-editing-using-the-alt-r-crispr-system-in-em-c.-elegans-em) and included 15.5 μM tracrRNA, 14.3 μM target crRNA, 5 μM *dpy-10* crRNA, 7 μM Cas9 protein, 6 μM target repair template and 0.5 μM *dpy-10* repair template. To prepare the mix, the tracrRNA and crRNA were incubated at 95°C for 5 min then 20°C for 5 min to hybridise the gRNA complex. Cas9 was incubated with the gRNA at 37°C for 15 min to form the ribonucleoprotein complex. The target and *dpy-10* repair templates were then added at room temperature. Mixes were injected into the gonads of young adult animals. Injected animals were grown at 20°C or 25°C and offspring were screened at L4-adulthood for the dpy or rol phenotypes. Dpy/rol animals were genotyped by PCR and restriction enzyme digest to identify successfully edited animals. Correct editing was confirmed by Sanger sequencing. Edited strains were outcrossed 2–6 times to remove the *dpy* mutation and potential background mutations (as indicated in the key resources table). crRNA and repair template sequences are included in Table S2.

#### Brood size assays

Regular brood size: single L4 hermaphrodites were plated onto growth plates and transferred to new plates every 24 hours until they had stopped laying or died. After each animal was removed, plates were incubated for 24 hours then scored for the number of live progeny, unfertilised oocytes, and, for Figures 1B and 1C, dead eggs and dead L1s.

For the brood size assay including wild-type, *sdha-2(tm1420), icl-1(ok531),* and *sdha-2(tm1420); icl-1(ok531)* animals, all animals were derived from a single cross between *sdha-2(tm1420)* and *icl-1(ok531)* to ensure a consistent genetic background between strains. Three lines were selected from the single cross for each genotype, used in three independent brood size assays (N = 8 worms per assay, 24 total).

Testing for a defect in hermaphrodite self-sperm: *sdha-2(tm1420)*, *sdha-2(smb65)* or wild-type L4 hermaphrodites were mated to wild-type L4 males for 24 hours. Males were then removed, and hermaphrodites transferred to new plates every 24 hours until they had stopped laying or died. Live progeny were scored as above. The percentage of males was monitored, and approximately 50% males were produced from every hermaphrodite parent, indicating successful matings.

Testing for a defect in male sperm: wild-type L4 hermaphrodites were mated to *him-8(e1489)*, *sdha-2(tm1420); him-8(e1489)*, AKA36; *him-8(e1489)*, or AKA36 with repaired *sdha-2; him-8(e1489)* L4 males each carrying an integrated *glr-1::gfp* neuronal reporter transgene for 24 hours. Males were then removed, and hermaphrodites transferred to new plates every 24 hours until they had stopped laying or died. The number of GFP-positive (cross-progeny) and -negative (self-progeny) live progeny were scored as above. The vast majority of *him-8(e1489)* and AKA36 with repaired *sdha-2; him-8(e1489)* animals produced predominantly GFP-positive (cross) progeny. Two animals from each strain produced five or less GFP-positive offspring indicating failed mating, and these data points were removed as outliers.

Each experiment was performed with n = 10–12 animals at Day 0, except for Figure 1B where n = 5, and Figures 4E–4H where n = 24. Animals which died or were lost within the first three days of adulthood were excluded from analysis. Scoring was performed blind to the strain genotype.

#### SDHA-2 protein structure prediction and alignment

The protein structure of *C. elegans* SDHA-2 has not been elucidated, so we modelled SDHA-2 using the 3D protein structure prediction tool I-TASSER.^21^ The entire SDHA-2 protein sequence was submitted to protein structure and function prediction tool I-TASSER https://zhanglab.ccmb.med.umich.edu/I-TASSER/.^21^ The top structural analogue identified for SDHA-2 was PDB: 1YQ3A, subunit A of Avian respiratory complex II with oxaloacetate and ubiquinone.^22^ The modelled SDHA-2 structure obtained from I-TASSER was aligned to 1YQ3A (subunit A only) (96% residues aligned with 72% sequence identity) or 1YQ3 (all 4 subunits) in PyMOL (Schrödinger). The top SDHA-2 model compared with 1YQ3 gave a TM-score of 0.958 and RMSD of 0.33. SDHA-2 residues predicted to be directly involved in binding the malate-like intermediate ligand were identified by homology to Avian respiratory complex II subunit A^22^ and comprise Gly79, Gln268, His270, Thr282, Glu283, Arg314, His381, Arg426, Ala429. All 9 residues were conserved between the two proteins. Structure images were exported from PyMOL.

#### *In vitro* sperm activation assay

Sperm activation assays were performed as follows.^84^ *him-8(e1489)*, AKA36; *him-8(e1489)* and *sdha-2(tm1420); him-8(e1489)* L4 males were isolated and allowed to mature to adulthood in the absence of hermaphrodites for 24 hours to acquire virgin day 1 adult males. Virgin males were dissected near the tail in sperm medium (50 mM HEPES, 25 mM KCl, 45 mM NaCl, 1 mM MgSO_4_, 5 mM CaCl_2_, 10 mM Dextrose; pH 7.8), or sperm medium with 200 μg/mL Pronase (Sigma, P5147) or 0.1 μm Monensin (Sigma) to release spermatids on glass slides. Dissected spermatids were incubated for 45 min before imaging (pronase assay), imaged every 30 second for 1 hr post dissection (monensin assay) or imaged every 1 min for 40 min post dissection (NAC supplementation assay).

#### *In vivo* sperm motility assays

One day before the assay L4 males were isolated and allowed to mature to adulthood on MitoTracker Red (MTR) containing plates. Plates were prepared by pipetting a dilution of 5 μL of 1mM MTR in 50 μL of M9 buffer onto the OP50 lawn and allowing it to dry. Males were grown overnight at 20°C in the dark. 1 hour before mating, males were moved to a recovery plate without MTR. Hermaphrodites were immobilised (0.1% tetramisole for 5–10 min) and then added to mating plates with ∼50 males for 30 min). Half of the hermaphrodites were immediately imaged every 30 sec on a Nikon C2 Basic Confocal microscope with a 100X oil immersion objective for sperm tracking analysis. The other half of the hermaphrodites were imaged 1 hr after mating for zone localisation analysis. The experiment was repeated twice. Tracking analysis and zone localisation analysis was performed as described in Hu et al.^24^ Briefly, sperm were tracked using the TrackMate plugin in ImageJ.^85^ The uterus was divided into thirds (zone 1 closest to the vulva, zone 3 closest to the spermatheca) and only those sperm visible in zone 2 were tracked. The same zones were used to classify sperm distribution throughout the uterus in the 1 hr post-mating images. Only animals where sperm were distinct enough to count were classified.

#### Oxygen consumption rate (OCR) seahorse assay

OCR of live animals was measured using an Agilent Seahorse XF24 Analyzer (Agilent Technologies) according to the manufacturer’s instructions, and adapted from Koopman et al.^86^ L4 hermaphrodites were incubated for 24 hours on culture plates. The next day, the day one adults were collected and washed in M9. After 30 minutes had elapsed to allow bacteria to have cleared from the gut, ∼20 animals were plated into each well of a 24-well islet capture microplate (Agilent Technologies). The precise number of animals per well was counted for subsequent data correction. Each plate consisted of 10 wells of wild-type, 10 of *sdha-2(tm1420)*, and 4 blank wells. Islet capture screens were fitted into each well, and M9 added to a total of 500 μL per well. OCR was measured in M9 under basal conditions (basal respiration), in response to the mitochondrial uncoupler carbonylcyanide-4-(trifluoromethoxy)-phenylhydrazone (FCCP) (0.01 mM final concentration, diluted in M9 from a 5 mM in DMSO stock) (maximal respiration), and the complex IV and V inhibitor sodium azide (40 mM final concentration) (non-mitochondrial OCR). OCR was measured for 2 min per loop, for 5 loops under basal conditions, 9 loops after FCCP addition, and 4 loops after sodium azide addition. Three independent experiments were performed, giving a total of 30 replicates each for wild-type and *sdha-2(tm1420)*. Raw OCR readings were divided by the number of animals per well to give corrected OCR in pMol/min/animal. Wells displaying very low OCR readings (<2 pMol/min/animal for whole experiment) (N = 1 for wild-type, N = 2 for mutant) or no OCR increase following FCCP addition (N = 1 for wild-type, N = 2 for mutant) were excluded from analysis. To generate basal, maximal, and non-mitochondrial respiration summary data, the means of the measurements from the final three loops of each condition were calculated.

#### Microscopy

To determine whether *sdha-2* males have defective sperm, GFP-positive and -negative animals were scored with a Nikon SMZ18 Microscope with Nikon Intensilight C-HGF1 Lamp.

In sperm morphology and pronase activation assays, brightfield microscopy was performed on mounted sperm using an Olympus BX51 Microscope fitted with an Olympus F-View II camera. Images were captured with AnalySIS software. Sperm were manually categorised as spermatids, spermatids with small protrusion, spermatozoa with pseudopod, and abnormal (spiked, vacuolated, or swollen). Sperm morphology metrics were quantified using ImageJ: sperm cells in brightfield images were manually outlined, and then shape metrics (area, circularity, solidity, aspect ratio) calculated using the ‘analyze particles’ tool.

For the NAC supplementation and monensin activation assays, timelapse DIC microscopy was performed using a Nikon ECLIPSE Ni-E Microscope. Sperm were manually categorised as above.

To visualise DAPI-stained sperm in hermaphrodites, L4 hermaphrodites were incubated for 24 hours to obtain day 1 adults. Animals were fixed in 3 × 95% ethanol as previously described^87^ and DNA was visualised with DAPI (300 ng/mL). DIC and fluorescent imaging was performed using a Nikon C2 Basic Confocal microscope. A Z-stack of images of each spermatheca was collected and flattened to create a maximum intensity projection image using ImageJ. Sperm from one spermatheca per hermaphrodite were counted as previously described.^88^

#### Mitochondrial membrane potential and morphology

To assess mitochondrial membrane potential, number and morphology, isolated spermatids were stained with JC-1 dye.^36^ Briefly, virgin males (prepared as in sperm activation assays) were dissected in sperm medium (50 mM HEPES, 25 mM KCl, 50 mM NaCl, 1 mM MgSO_4_, 5 mM CaCl_2_, 1 mg/mL BSA; pH 7.8) with 15 μM final concentration JC-1 (Invitrogen) to release spermatids on glass slides. After incubation at room temperature for 10 minutes, spermatids were washed three times with sperm medium then imaged in a single focal plane using a Nikon C2 Basic Confocal microscope. Strains were imaged under identical parameters, using the 60x objective (mitochondria morphology) or 100x objective (red:green ratio).

JC-1 red:green ratio was quantified using ImageJ as follows. First, a binary mask of spermatids was generated. A generous intensity-based threshold was applied to the green channel and converted to a binary mask. The mask was size filtered to remove small particles. The ‘fill holes’ command and two rounds of ‘dilute’ and ‘erode’ were applied so the mask encapsulated whole spermatids. Watershed was applied to separate neighbouring spermatids. For quality control, the mask was converted to outlines and overlaid over the original image to confirm accuracy. The mask was then used to determine the total fluorescence intensity in each spermatid in the red and green channels, and the red:green ratio was calculated for each spermatid.

Mitochondrial morphology parameters in JC-1-stained spermatids were quantified using ImageJ as follows, adapted from de Boer et al.^89^ Images were pre-processed by background subtraction and the Enhance Local Contrast (CLAHE) tool. The Laplacian tool of the FeatureJ plugin was applied (highlights regions of rapid intensity change, used for edge detection), and then converted to a binary mask by thresholding (IsoData). The mask was filtered by pixel size to remove noise/debris (≥7 pixels retained), and then analysed for shape metrics: area, circularity, aspect ratio and Feret’s diameter. The analysis was performed on both the red and green channels.

The number of JC-1-stained mitochondria in a single focal plane per sperm cell in the red and green channels was quantified using ImageJ as follows. The mask outlining individual mitochondria from the morphology analysis was converted to points using ‘find maxima’, so that each mitochondrion was represented by a uniform one-pixel point. Spermatids were then outlined using the same approach as in the red:green ratio analysis, and the regions of interest applied to the mitochondria-as-points image. Total integrated density per sperm was then calculated and divided by 255 (the value of a single pixel) to yield the number of mitochondria per sperm. The analysis was performed on both the red and green channels.

#### Redox probe assays

Virgin males were prepared as for the sperm activation assays and dissected in 20 μL sperm medium containing 10 μM final concentration of NpFR1 or NpFR2 to release spermatids on glass slides. After incubation at room temperature for 10 min (NpFR1) or 40 min (NpFR2), spermatids were imaged in a single focal plane using a Nikon C2 confocal microscope. All images were taken under identical conditions using the 100x objective. Green fluorescence intensity was quantified using ImageJ as follows. First, a binary mask of spermatids was generated. An intensity-based threshold (Triangle method) was applied to the green channel and converted to a binary mask, and the ‘fill holes’ command applied. Watershed was applied to separate neighbouring spermatids. The mask was size filtered to remove small particles. Ellipses were auto-fitted to each spermatid using the ‘analyse particles’ function so that the mask encapsulated whole spermatids, and watershed applied again. For quality control, the mask was converted to outlines and overlaid over the original image to confirm accuracy. The mask was then used to determine the mean fluorescence intensity in each spermatid.

#### UPR_mt_ and oxidative stress assays

To image animals expressing GFP reporters of UPR_mt_ and oxidative stress, L4 or day 1 adult animals were immobilised with 0.2% Tetramizol in M9 buffer (22 mM KH_2_PO_4_, 50 mM Na_2_HPO_4_, 86 mM NaCl, 1 mM MgSO_4_) and mounted on glass slides. DIC and fluorescent imaging was performed using standard methods using an Olympus BX51 Microscope fitted with an Olympus F-View II camera. Images were captured with AnalySIS software. For each reporter, wild-type and *sdha-2(tm1420)* strains were imaged under identical parameters with consistent focal planes between individuals. Quantification was performed using ImageJ as follows: for each image, the area of the whole animal was selected using the polygon selection tool and mean grey value (representing fluorescence intensity) measured. Mean grey value was also measured for a portion of the image background. The adjusted fluorescence intensity was calculated (mean grey value of animal – average mean grey value of all background readings), then normalised to wild-type to give relative fluorescence intensity.

### Quantification and statistical analysis

Number of animals used in experiments and statistical tests are indicated in figure legends. For all analyses except RNAseq, statistical analyses as indicated in figure legends were performed using GraphPad Prism. Statistical significance was defined as p < 0.05. Error bars indicate standard error of the mean (SEM) as stated in figure legends.

Microscopy analysis was performed in Fiji (ImageJ) and is described in the corresponding STAR methods section.

#### Whole genome sequencing analysis and mapping of non-synonymous mutations

Data QC, processing and analyses were performed using CLC Genomics (QIAGEN). Average coverage was ∼70x. Libraries were filtered to remove short reads <15, and a quality limit of 0.01 applied. Reads were mapped to WormBase release WS260. Mutations present in AKA36 and absent in SX461 were identified using the “identify known mutations from sample mappings” tool. Mutations were filtered to identify functional variants, and then non-synonymous mutations on Chromosome I.

#### RNAseq analysis

RNAseq data from 12 environmental stress conditions from 5 different published datasets were analysed (see table below). Analysis was performed using R version 4.1.0 and RStudio version 1.4.1717. Fastq files from each stress dataset were downloaded from SRA using fasterq-dump. Adaptors were removed and files were quality trimmed using Cutadapt software.^73^ Read alignment and counting were performed using STAR aligner allowing for zero mismatches.^74^ The DESeq2 R package^75^ was used to obtain normalised count data and identify differentially expressed genes using a filter threshold of adjusted *q* value <0.05. Pre-filtering was performed to remove genes with no expression data. Gene set enrichment analysis was done using the GSEA standalone software (Broad Institute,^76^), with manually curated gene lists derived from GO term lists and Wormpaths^90^ pathway lists. The GSEA parameters used were signal to noise ranking on uncollapsed data with 20,000 permutations. The ETC list contained the GO terms: electron transport chain (GO:0022900) and oxidative phosphorylation (GO:0006119) as well as the Wormpaths ETC list and two added complex II assembly factors (F25H9.7 and W02D3.12) that were absent from these lists. The TCA cycle list contained the tricarboxylic acid cycle (GO0006099), the Wormpaths TCA list and the two complex II assembly factors (F25H9.7 and W02D3.12). The mitochondria list was a combined list of the ETC and TCA cycle list as well as the GO term mitochondrion (GO:0005739) with duplicates removed. Graphs were plotted using the ggplot2 and ComplexHeatmap packages.^77^ KEGG analysis was performed using ShinyGO^78^ with a FDR cutoff of 0.05 and plotted with ggplot2.

**Table:** RNAseq stress conditions

Stress name	Method	Source
1hr–16hr Starved	L4 worms were washed onto unseeded plates and left for the indicated time.	Harvald et al.^91^
Heat shock	Synchronised L1 worms were placed on HT115 bacteria containing the empty L4440 plasmid. Heat shock was performed at the L4 stage for 30 minutes in a 33C water bath.	Brunquell, J. et al.^92^
Post-dauer Starved	Starvation-induced post-dauer adults. Worms were placed on OP50 plates and left to starve. 1% SDS was used to select for dauers which were then transferred to fresh plates fed as normal until they were day 1 adults.	Ow et al.^93^
Rotenone	L4 worms were washed onto heat inactivated OP50 plates with 100nm Rotenone, an ETC complex I inhibitor, and left for 24 hours.	Schmeisser et al.^94^
Pathogen	Embryos isolated from bleached adult hermaphrodites were raised on growth plates with L4440 bacteria until the L4/young adult transition stage, then transferred to OP50 or PA14 bacteria plates for 6 hours.	Ding et al.^95^

## Data Availability

This paper analyses existing, publicly available data. The accession numbers for these datasets are listed in the key resources table. All other data reported in this paper will be shared by the lead contact upon request. This paper does not report original code. Any additional information required to reanalyse the data reported in this paper is available from the lead contact upon request.

## Supplemental information

Document S1. Figures S1–S6 and Tables S1 and S2

Data S1. Raw differential expression data for all genes tested in each stress condition, related to Figure 8

Data S2. Hierarchical clustering gene sets with expression data from each stress condition, related to Figure 8
