# Supplementary material for: Mitochondrial succinate dehydrogenase function is essential for sperm motility and male fertility
Source: iScience. 2022 Nov 14;25(12):105573. doi: 10.1016/j.isci.2022.105573 (PMC9709242; doi:10.1016/j.isci.2022.105573)
Supplement: Document S1. Figures S1–S6 and Tables S1 and S2 [file mmc1.pdf]

iScience, Volume *25*

Supplemental information

Mitochondrial succinate dehydrogenase function

is essential for sperm motility and male fertility

Rachel M. Woodhouse, Natalya Frolows, Guoqiang Wang, Azelle Hawdon, Edmund Heng Kin Wong, Linda C. Dansereau, Yingying Su, Liam D. Adair, Elizabeth J. New, Ashleigh M. Philp, Wei Kang Tan, Andrew Philp, and Alyson Ashe

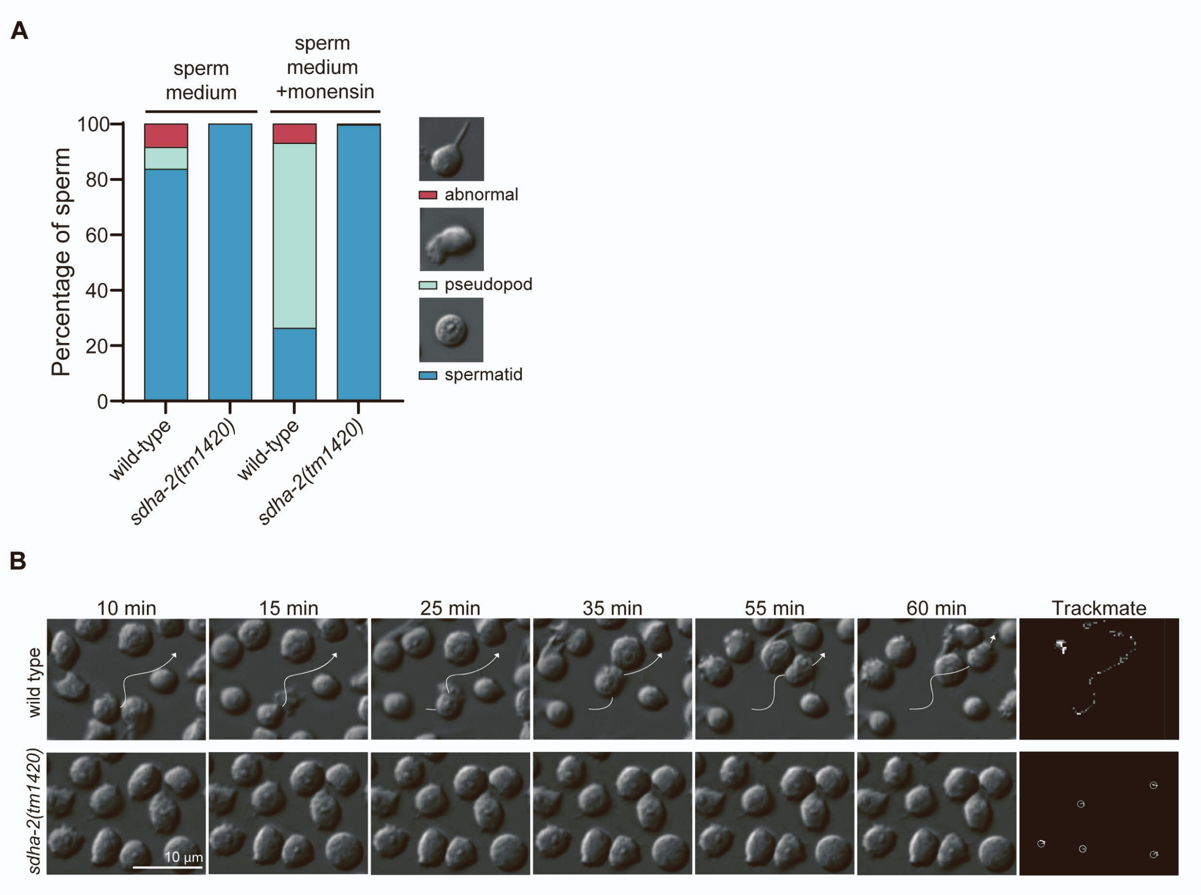

Figure S1: *In vitro* sperm activation with monensin, related to Figure 4. (A) Virgin adult males were dissected in sperm medium with or without monensin, as indicated. n ≥180 sperm from ≥5 males. (B) Representative images of wild-type and *sdha-2* mutant sperm in 0.1 µM monensin. Scale bar = 10 µm.

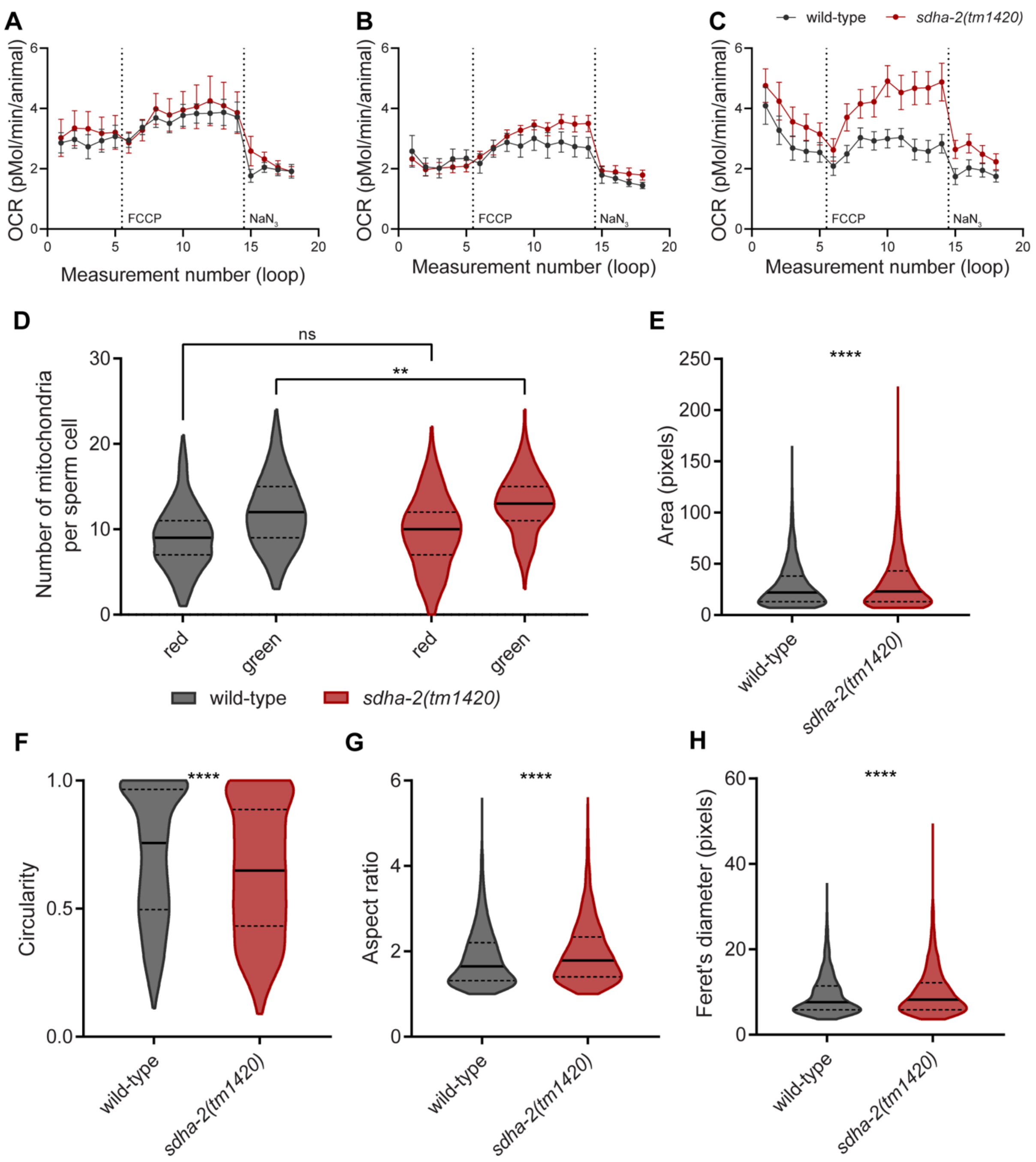

Figure S2: *sdha-2* mutant sperm display aberrant mitochondrial morphology and number in JC-1 green channel, related to Figure 6. (A-C) Three independent experiments assessing oxygen consumption rate (OCR) in live day 1 adult hermaphrodites using a Seahorse Analyzer. OCR was measured in M9 under basal conditions (basal respiration), in response to the mitochondrial uncoupler carbonylcyanide-4-(trifluoromethoxy)-phenylhydrazone (FCCP) (maximal respiration), and the complex IV and V inhibitor sodium azide (NaN_3) (non-mitochondrial OCR). Compound additions

are marked by dotted lines. Raw OCR readings were divided by the number of animals per well to give corrected OCR in pMol/min/animal. Data are mean ± SEM; n=6-10 in each experiment. (D) The number of JC-1-stained mitochondria in a single focal plane per sperm cell in the red or green channel. n = 352 wild-type sperm and 144 *sdha-2(tm1420)* sperm. Comparisons were performed using two-way ANOVA with Sidak's post hoc test **$p \leq 0.01$. Violin plots display the median (solid line) and quartiles (dashed lines). (E-H) Sperm were stained with JC-1 dye, and mitochondrial morphology parameters in the green channel were calculated using ImageJ, including (E) area, (F) circularity (where 1 is a perfect circle), (G) aspect ratio (major axis/minor axis) and (H) Feret's diameter (the longest distance between any two points in an object). n = 4197 mitochondria for wild-type and 1966 for *sdha-2(tm1420)*. Comparisons were performed using unpaired t-tests. ****$p \leq 0.0001$. Note that strains carried the *him-8(e1489)* mutation, which increases the prevalence of males in the population for ease of male isolation without altering the quality of sperm.

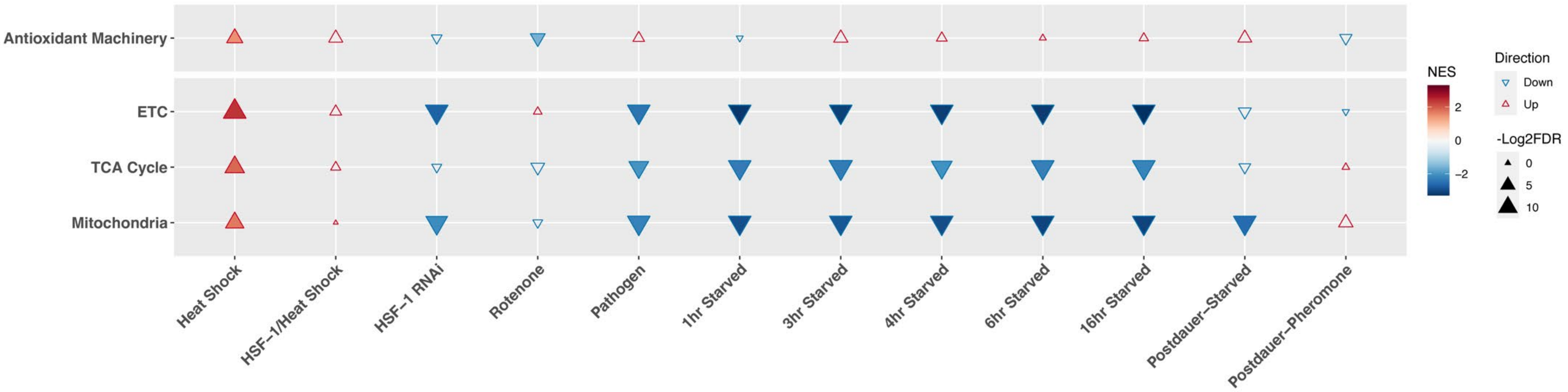

Figure S3: GSEA results of all 12 datasets tested, related to Figure 8. Gene set enrichment analysis (GSEA) of mRNA transcripts after each stress exposure. A positive normalised enrichment score (NES) indicates that the gene set was upregulated after stress exposure. Hollow triangles indicate a lack of statistical significance (FDR>0.05, -Log2(FDR)<4.3). Gene sets were based on GO term categories and manually curated for greater accuracy, see STAR Methods for details.

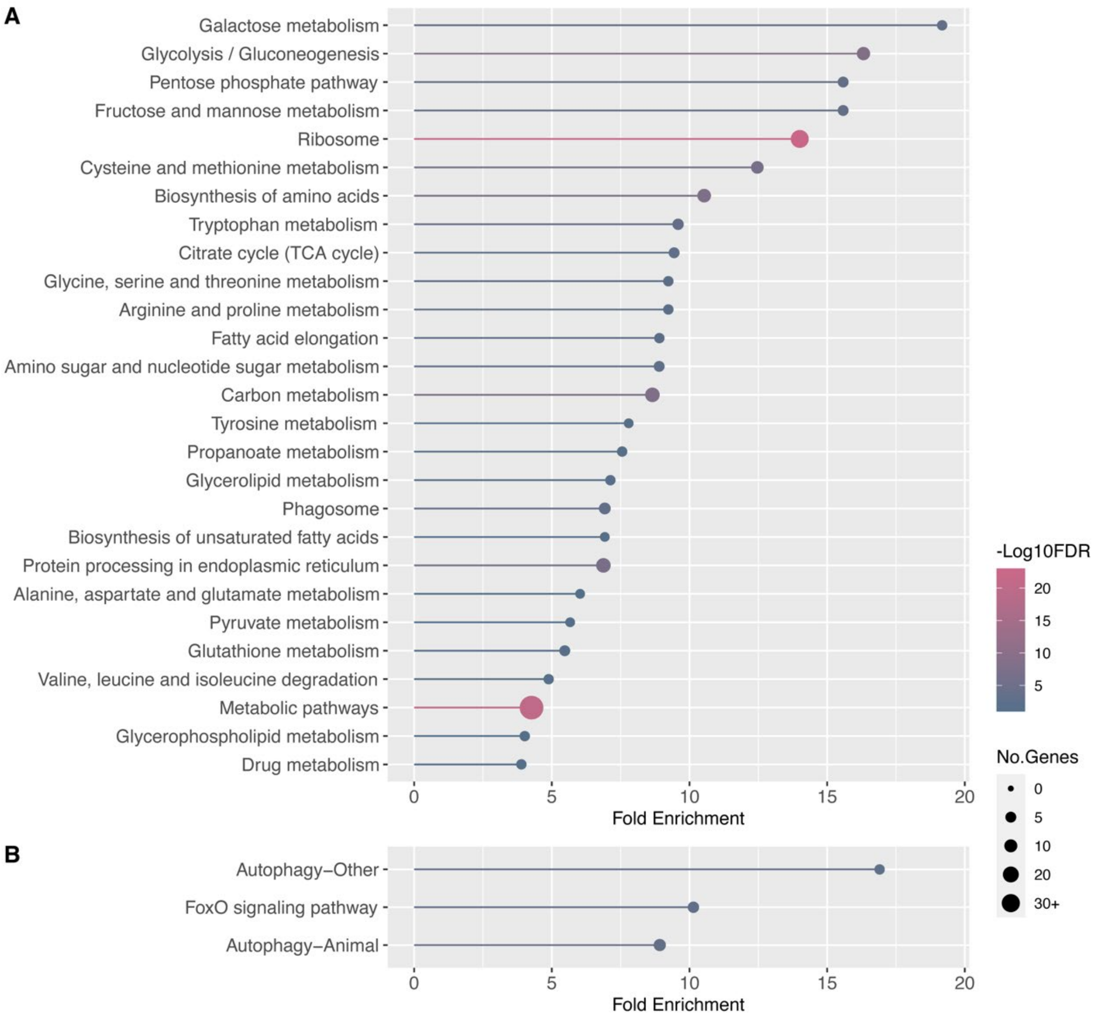

Figure S4: KEGG pathway analysis, related to Figure 8. Significantly enriched KEGG pathways from (A) Class I genes and (B) Class II genes. Gene sets were extracted from the hierarchical clustering in Figure 8D and analysed using ShinyGO, outputs were plotted with ggplot2.

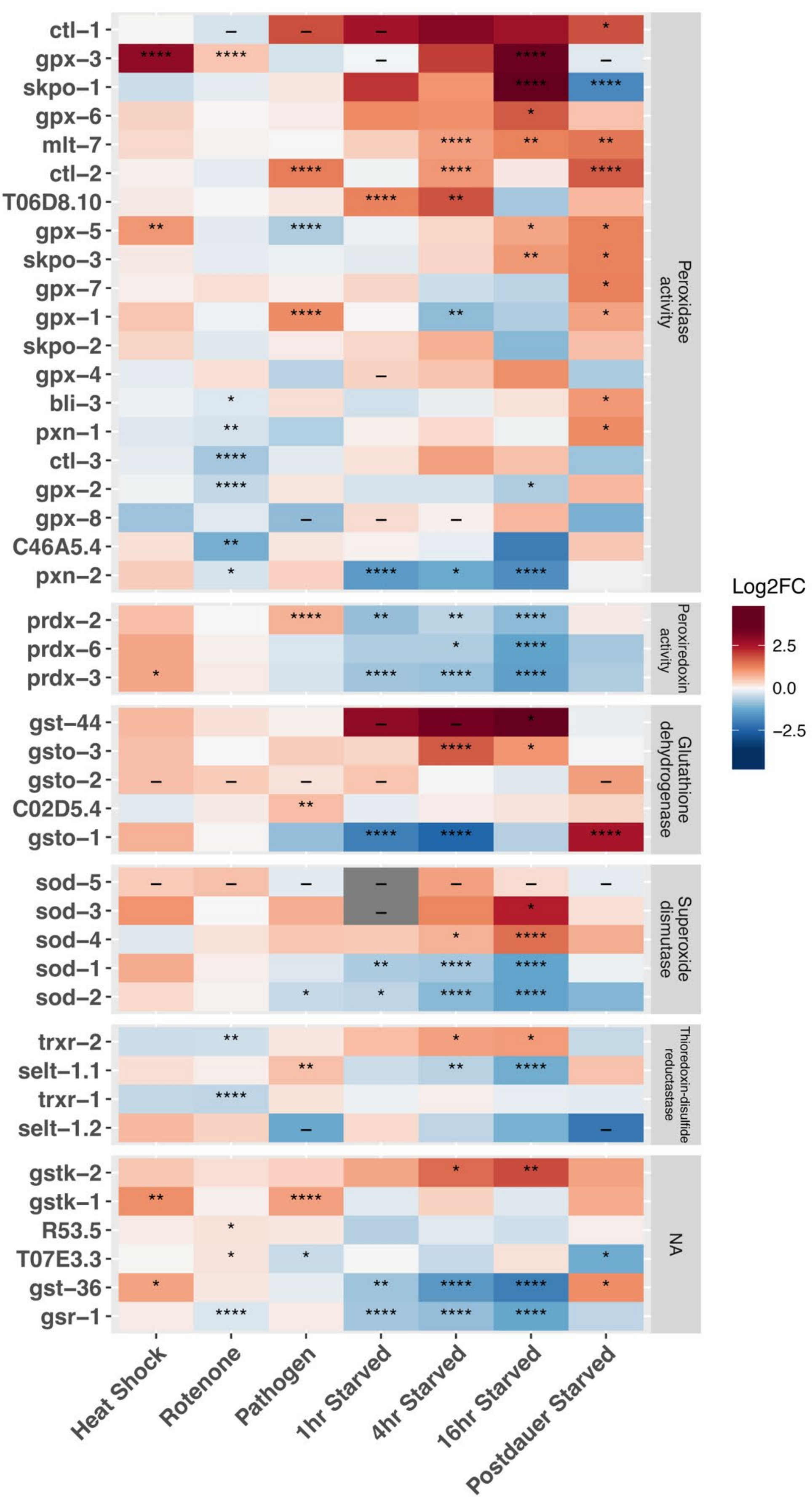

ctl−1
gpx−3
skpo−1
gpx−6
mlt−7
ctl−2
T06D8.10
gpx−5
skpo−3
gpx−7
gpx−1
skpo−2
gpx−4
bli−3
pxn−1
ctl−3
gpx−2
gpx−8
C46A5.4
pxn−2
Peroxidase activity
prdx−2
prdx−6
prdx−3
Peroxiredoxin activity
gst−44
gsto−3
gsto−2
C02D5.4
gsto−1
Glutathione dehydrogenase
sod−5
sod−3
sod−4
sod−1
sod−2
Superoxide dismutase
trxr−2
selt−1.1
trxr−1
selt−1.2
Thioredoxin-disulfide reductastase
gstk−2
gstk−1
R53.5
T07E3.3
gst−36
gsr−1
NA
Heat Shock
Rotenone
Pathogen
1hr Starved
4hr Starved
16hr Starved
Postdauer Starved
Log2FC
2.5
0.0
−2.5

Figure S5: Heatmap of antioxidant activity (GO:0016209) genes, related to Figure 8. Genes were separated into the descendant GO term categories: antioxidant activity (GO:0016209), glutathione dehydrogenase (ascorbate) activity (GO:0045174), glutathione-disulfide reductase (NADPH) activity (GO:0004362), peroxidase activity (GO:0004601), peroxiredoxin activity (GO:0051920), superoxide dismutase activity (GO:0004784) and thioredoxin-disulfide reductase activity (GO:0004791). Dashes indicate an NA value.

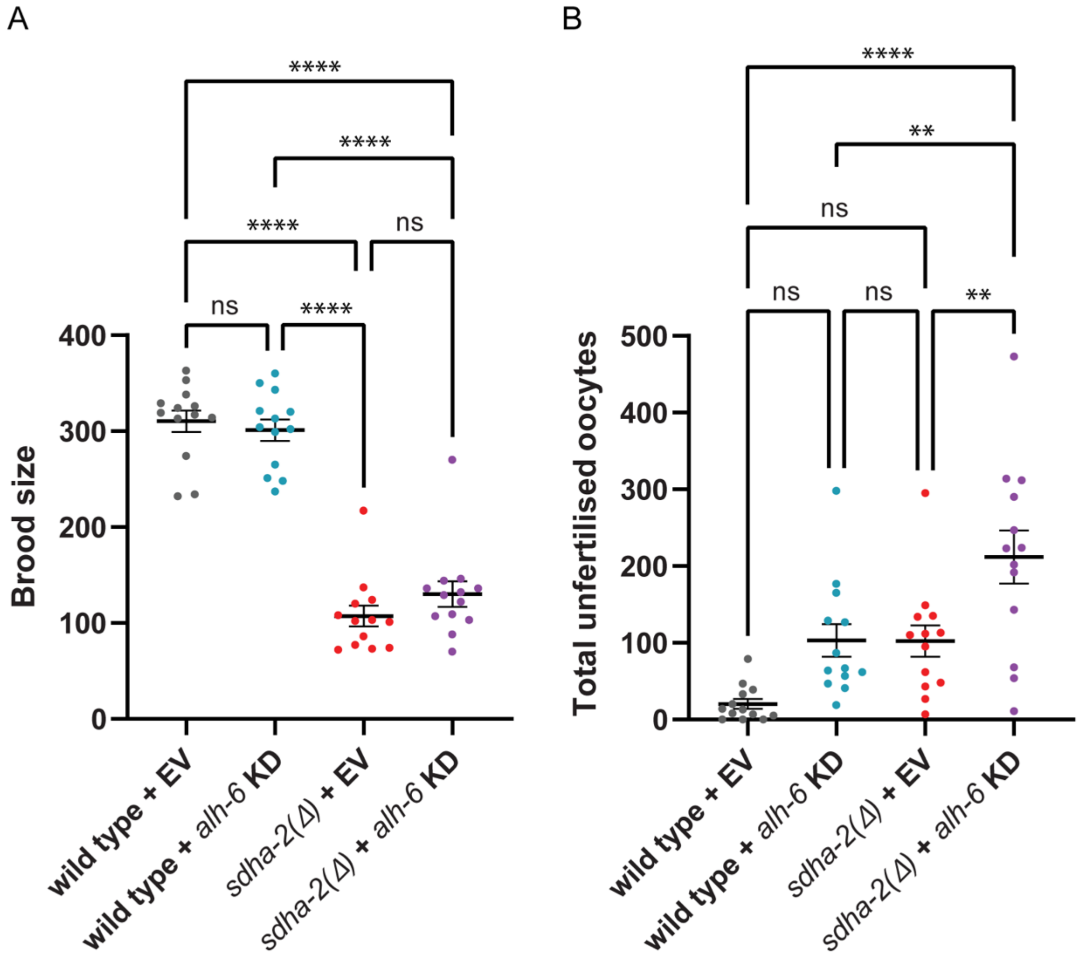

Figure S6: Broodsize assay of *alh-6* knockdown fertility, related to Figure 8. The total number of (A) live progeny and (B) unfertilised oocytes per animal. The experiment was repeated on 3 different occasions with n=4-5 animals per assay. Data are mean ± SEM; n = 13. Comparisons were performed using one-way ANOVA with Tukey's post hoc test **p ≤ 0.01, ****p ≤ 0.0001.

Table S1: Non-synonymous mutations in strain AKA36, related to Figure 1.

Gene name	Genomic region of mutation	Type	Reference genome*	Allele in AKA36*	Amino acid change in longest transcript	Gene on plus or minus strand
col-60	7105935	SNV	G	C	Gly191Arg	+
marc-3	9418848	SNV	C	T	Gly9Asp	–
F30A10.15	9482785..9482789	5 bp deletion	ATGTC	-	Removes start codon	+
set-32	9519410	SNV	C	T	Pro36Ser	+
set-32+	9519451..9519964	514 bp deletion	514 nucleotides	-	In-frame deletion of Ala50 – Ala205	+
nra-2	9622176	SNV	G	A	Val555Ile	+
tba-1	9785983	SNV	C	T	Gly439Arg	–
mgl-2	10387704	SNV	G	A	Gln538Stop	–
sdha-2	10684606	SNV	C	T	Gly278Glu	–
vab-10	11747915	SNV	C	T	Arg5041Lys	–

SNV = single nucleotide variation

*Reported on the plus strand of the *C. elegans* reference genome Wormbase Genome Browser version WS275

+Previously reported *set-32(ok1457)* allele

Table S2: Sequences of CRISPR guides and repair templates used in this study, related to STAR Methods.

Target	Allele created	Strain created	Guide sequence (PAM in brackets) (5'-3')	Repair sequence (5'-3' on protospacer strand)
dpy-10	*cn64* (Arribere et al., 2014)	n/a	GCTACCATAGGCACCACGAG(CGG)	CACTTGAACTTCAATACGGCAAGATGAGAATGACTGGAAACCGTACCGCATGCGGTGCCTATGGTAGCGGAGCTTCACATGGCTTCAGACCAACAGCCTAT
marc-3	*smb53*	AKA157	CAACGCATCGCTGGGTCCAG(CGG)	GGTCGAGGCAATGGAAGACTTCAACGCATCGCTGGATCCAGCTGTGTGTCGGATATGTATGTGTGGCGAGACTTCAATT
F30A10.15	*smb55*	AKA156	CATTTTGAATGGAATGAATG(CGG)	TTATAGAAAAATGCTAGTAATAGGTGAGTTGGTTTTGAATGGAATGAATTCAGGAATCAATACATTTGAACCAACGTGCAATGAGGAGAGAAGGTGATGACAA
sdha-2	Repairing *smb65* to wildtype sequence	AKA145	GAAGGATCACGAGGAGAGGG(TGG)	TTCTGATATGGAGTTTGTTCAATTCCATCCAACTGGAATCTACGGAGTTGGATGTTTGATCACCGAAGGATCCCGTGGCGAAGGTGGATATTTGGTCAATTCGCAAGGAGAACGATTTATGGAAAGATATGCACCGA
sdha-2	*smb65*	AKA199	GAAGGATCACGAGGAGAGGG(TGG)	TTCTGATATGGAGTTTGTTCAATTCCATCCAACTGGAATCTACGGAGTTGAATGTTTGATCACCGAAGGATCCCGTGGCGAAGGTGGATATTTGGTCAATTCGCAAGGAGAACGATTTATGGAAAGATATGCACCGA
